# Programmed surface on poly(aryl-ether-ether-ketone) initiating immune mediation and fulfilling bone regeneration sequentially

**DOI:** 10.1016/j.xinn.2021.100148

**Published:** 2021-08-05

**Authors:** Lingxia Xie, Guomin Wang, Yuzheng Wu, Qing Liao, Shi Mo, Xiaoxue Ren, Liping Tong, Wei Zhang, Min Guan, Haobo Pan, Paul K. Chu, Huaiyu Wang

**Affiliations:** 1 Center for Human Tissues and Organs Degeneration, Shenzhen Institute of Advanced Technology, Chinese Academy of Sciences, Shenzhen 518055, China; 2 Department of Physics, Department of Materials Science and Engineering, and Department of Biomedical Engineering, City University of Hong Kong, Tat Chee Avenue, Kowloon, Hong Kong, China; 3 Technical Institute of Physics and Chemistry, Chinese Academy of Sciences, Beijing 100190, China

**Keywords:** poly(aryl-ether-ether-ketone), surface modifications, sequential release, immune-mediated osteogenesis, bone regeneration

## Abstract

The immune responses are involved in every stage after implantation but the reported immune-regulated materials only work at the beginning without fully considering the different phases of bone healing. Here, poly(aryl-ether-ether-ketone) (PEEK) is coated with a programmed surface, which rapidly releases interleukin-10 (IL-10) in the first week and slowly delivers dexamethasone (DEX) up to 4 weeks. Owing to the synergistic effects of IL-10 and DEX, an aptly weak inflammation is triggered within the first week, followed by significant M2 polarization of macrophages and upregulation of the autophagy-related factors. The suitable immunomodulatory activities pave the way for osteogenesis and the steady release of DEX facilitates bone regeneration thereafter. The sequential immune-mediated process is also validated by an 8-week implementation on a rat model. This is the first attempt to construct implants by taking advantage of both immune-mediated modulation and sequential regulation spanning all bone regeneration phases, which provides insights into the fabrication of advanced biomaterials for tissue engineering and immunological therapeutics.

## Public summary

• A programed surface is designed and fabricated for immune-mediated osteogenesis
• The degradation of PTMC coating enables a sequential release of IL-10 and DEX
• Initially, osteoimmunomodulation is achieved by IL-10 and a small amount of DEX
• Afterwards, sustained release of DEX fosters the peri-implant bone regeneration

## Graphical abstract

**Figure:**
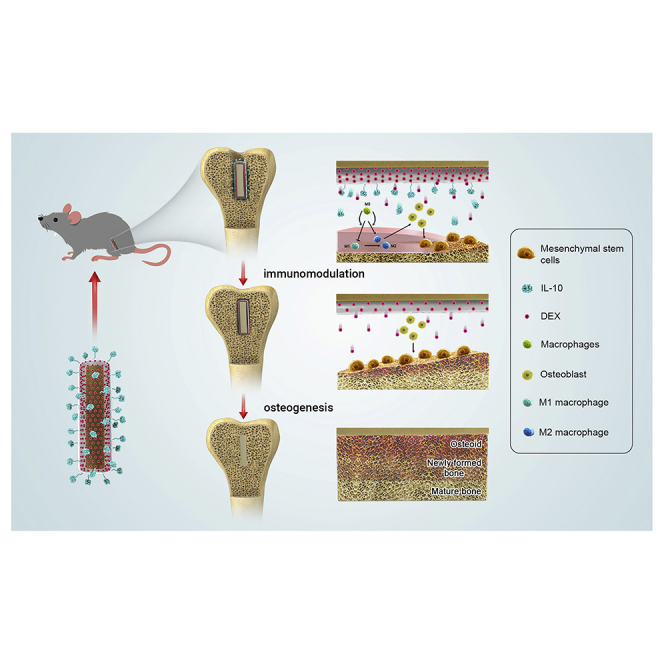

## Introduction

Long-lasting implants that can induce bone remodeling are highly expected to obviate the second surgery arising from unsuccessful bone regeneration.^1^, ^2^, ^3^ However, previous research activities have mainly modified the mechanical and biochemical properties of artificial implants including poly(aryl-ether-ether-ketone) (PEEK), but success *in vitro* may not preclude the excessive inflammation and/or poor bone integration hindering *in vivo* realization.^4^, ^5^, ^6^ The inconsistency between *in vitro* and *in vivo* experiments mainly stems from insufficient consideration of the whole osteogenesis process, which is impacted by multiple factors in the human body.^7^, ^8^, ^9^ Hence, the design of smart bone-implant materials based on an in-depth insight of bone regeneration will work more efficiently than the regulation of individual attributes using a trial-and-error method.^10^

Recently, a better understanding of the bone regeneration process after surgical implantation has been obtained.^11^^,^^12^ The consensus is that bone regeneration after implantation is a dynamic process which comprises the different phases of inflammation, bone formation, and bone remodeling that are impacted by the surrounding micro-environments.^10^^,^^13^^,^^14^ Within hours after implantation, the immune system is triggered with M1 macrophages secreting inflammation-related mediators and cytokines and small amounts of them are required for bone healing.^15^, ^16^, ^17^ Subsequently, smooth and timely polarization from M1 to M2 enables bone formation with matrix vascularization.^18^, ^19^, ^20^ Both the inflammation and subsequent transformation processes are pivotal but, in most cases, fibrotic capsules induced by the excessive accumulation of inflammatory factors compromise bone-implant osseointegration and increase the risk of implant failure.^20^^,^^21^ Although some biomaterials have been proposed to accelerate bone formation by interfering with the immune response, they primarily work during the very early stage after implantation but serious immunological rejection may be triggered afterward.^22^

On the molecular level, humanized interleukin-10 (IL-10) is a vital cytokine that helps macrophages adapt to the M2 phenotype and thus limit the inflammatory response *in vivo.*^23^, ^24^, ^25^ Besides, glucocorticoids are inherently anti-inflammatory and the steady and controlled release at a low dose can stimulate bone formation during the early weeks after implantation.^26^^,^^27^ Inspired by the conceptual molecular understanding of progressive bone formation, a surface co-functionalized with IL-10 and glucocorticoids that can be released orderly with the proper concentration is expected to tune M1-M2 polarization in the early inflammatory stage and promote osteogenetic differentiation thereafter. This can in turn create positive feedback by inhibiting inflammation to consequently foster bone formation. In this way, bone regeneration is accomplished by means of immune-mediated regulation. However, despite the prospect and potential, little effort was devoted to surface functionalization of bone implants that can program the peri-implant response in sequence for yielding the desirable immune-mediated regulation.

In this work, based on a comprehensive understanding of bone-implant interactions, humanized IL-10 and dexamethasone (DEX) (a well-used glucocorticoid) are synergistically utilized to modify PEEK implants to initiate the immunomodulation by cascade of IL-10 and a small amount of released DEX shortly after implantation. Subsequently, steady delivery of DEX during the following weeks allows smooth osteogenesis and bone formation throughout the process. By continuously building new bone with high quantity and quality, excellent bone remodeling can be accomplished *in vivo*. The programmed surface modification strategy that can promote bone regeneration by sequential regulation sheds light on the design of advanced biomedical implants.

## Results and discussion

### Sample characterization

**Figure 1:**
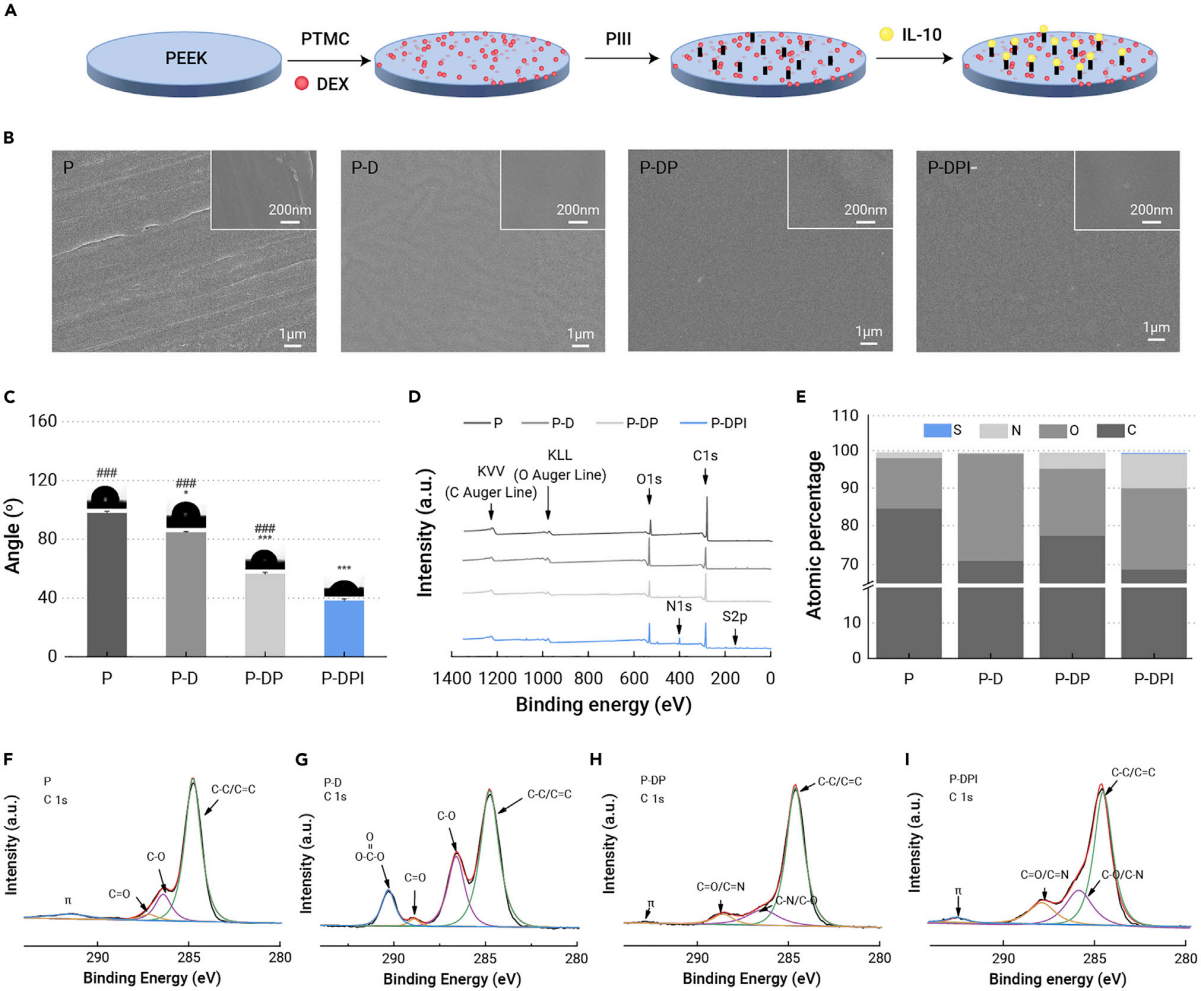
Sample fabrication and characterization (A) Flow chart showing the progress of sample fabrication. (B) SEM images showing the surface morphology of different samples. (C) Water contact angles of samples (n = 4). (D and E) (D) Survey XPS spectra as well as (E) atomic percentages determined on different samples. (F–I) High-resolution C 1s spectra of (F) P, (G) P-D, (H) P-DP, and (I) P-DPI samples. ∗p < 0.05 and ∗∗∗p < 0.001 compared with the P group, whereas ###p < 0.001 compared with the P-DPI group.

Figure 1A displays a flow chart illustrating the sample preparation process and the as-prepared samples were observed under scanning electron microscopy (SEM). The PEEK sample (defined as P) has a flat surface with minor scratches and, after addition of polytrimethylene carbonate (PTMC) and DEX (defined as P-D), fine dispersion was observed (Figure 1B). The PTMC/DEX coating was not impacted by N_2_ plasma immersion ion implantation (N_2_ PIII) (defined as P-DP), and the homogeneous topography after subsequent grafting of IL-10 (defined as P-DPI) suggests that IL-10 is uniformly introduced onto the surface. In addition, the fabricated coating was about 2.58 μm in thickness, which shows a sufficient binding with the PEEK substrate (Figure S1, supplemental information). As shown in Figure 1C, the bare PEEK substrate (P) is hydrophobic but the PTMC/DEX-coated sample (P-D) shows a smaller water contact angle due to the hydrophilic DEX molecules. The surface hydrophilicity of modified samples was further improved by N_2_ PIII treatment (P-DP), which on the other hand helps build the cohesion between the substrate and IL-10. Grafting of IL-10 reduces the water contact angle to 38° (P-DPI) and the resulting hydrophilic surface is expected to foster the attachment of osteoblasts.^28^

The chemical states on various samples are examined by X-ray photoelectron spectroscopy (XPS). Peaks for C 1s and O 1s emerged in all samples and a N 1s peak was observed after N_2_ PIII treatment (P-DP, Figure 1D). The enhanced N 1s and S 2p peaks detected from the P-DPI sample arise from the grafted IL-10. The effectiveness of surface modification in each step was verified by determining the percentages of different chemical groups (Figure 1E). The high-resolution C 1s spectrum shows C-C/C=C, C-O, and C=O peaks of the P sample (Figure 1F) and the peak at 290.3 eV of the P-D sample is associated with OOC=O in PTMC (Figure 1G). On the P-DP sample, nitrogen bonds with carbon forming C=N and C-N, but C=O/C-O decreases due to energetic plasma bombardment (Figure 1H). After grafting of IL-10, C=N and C-N of the P-DPI sample are more prominent, as shown in Figure 1I. The chemical changes were confirmed by the high-resolution spectra of N 1s, O 1s, and S 1s, as shown in Figure S2 (supplemental information). The characterization results above indicate the effectiveness of surface functionalization of PEEK.

### Release kinetics of IL-10 and DEX

**Figure 2:**
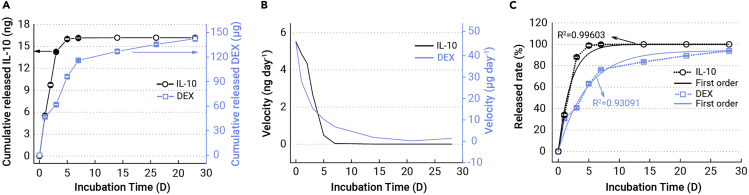
Release parameters of IL-10 and DEX of P-DPI samples in the solution containing lipase as a function of time (A) Cumulative released amounts. (B) Release velocities. (C) Percentages of cumulative release (symbols and dashed lines) as well as curves showing the first-order releasing kinetics (solid lines) (n = 4).

A series of tests were carried out to evaluate how biomolecules are released sequentially (Figure 2). With reference to previous studies,^29^^,^^30^ lipase was added to the solution to mimic the *in vivo* environment that triggers PTMC degradation. As shown in Figure 2A, a rapid release of IL-10 was detected from the P-DPI sample during the initial 5 days and more than 15 ng of IL-10 were released to the solution by the fifth day, constituting more than 90% of the total grafted amount (black symbols). This is also reflected by the release velocity which begins as high as 5 ng day^−1^ but falls precipitously to zero afterward (black line, Figure 2B). About 100% of the grafted IL-10 was released within 7 days, but the release curve of DEX is somewhat different from that of IL-10. In particular, PTMC containing DEX degrades gradually and 110 μg (60%) of DEX were dissolved in the solution during the first week (blue symbols, Figure 2A). Afterward, the remaining DEX was released at a slower pace and it took more than 4 weeks for the release rate to reach zero (blue line, Figure 2B). The data for these two molecules were fitted with different equations and the release curves obey first-order kinetics with coefficients of 0.99603 for IL-10 and 0.93091 for DEX (Figures 2C and S3, supplemental information). These two release curves are consistent with the intended design that IL-10 grafted on top is for primary control in the first few days and DEX coated with PTMC is maintained at an effective level for the next few weeks. As a result, IL-10 is supposed to trigger the M2 polarization of macrophages in the early stage and DEX can produce continuous effects to promote osteogenesis thereafter.

### Immunological response *in vitro*

**Figure 3:**
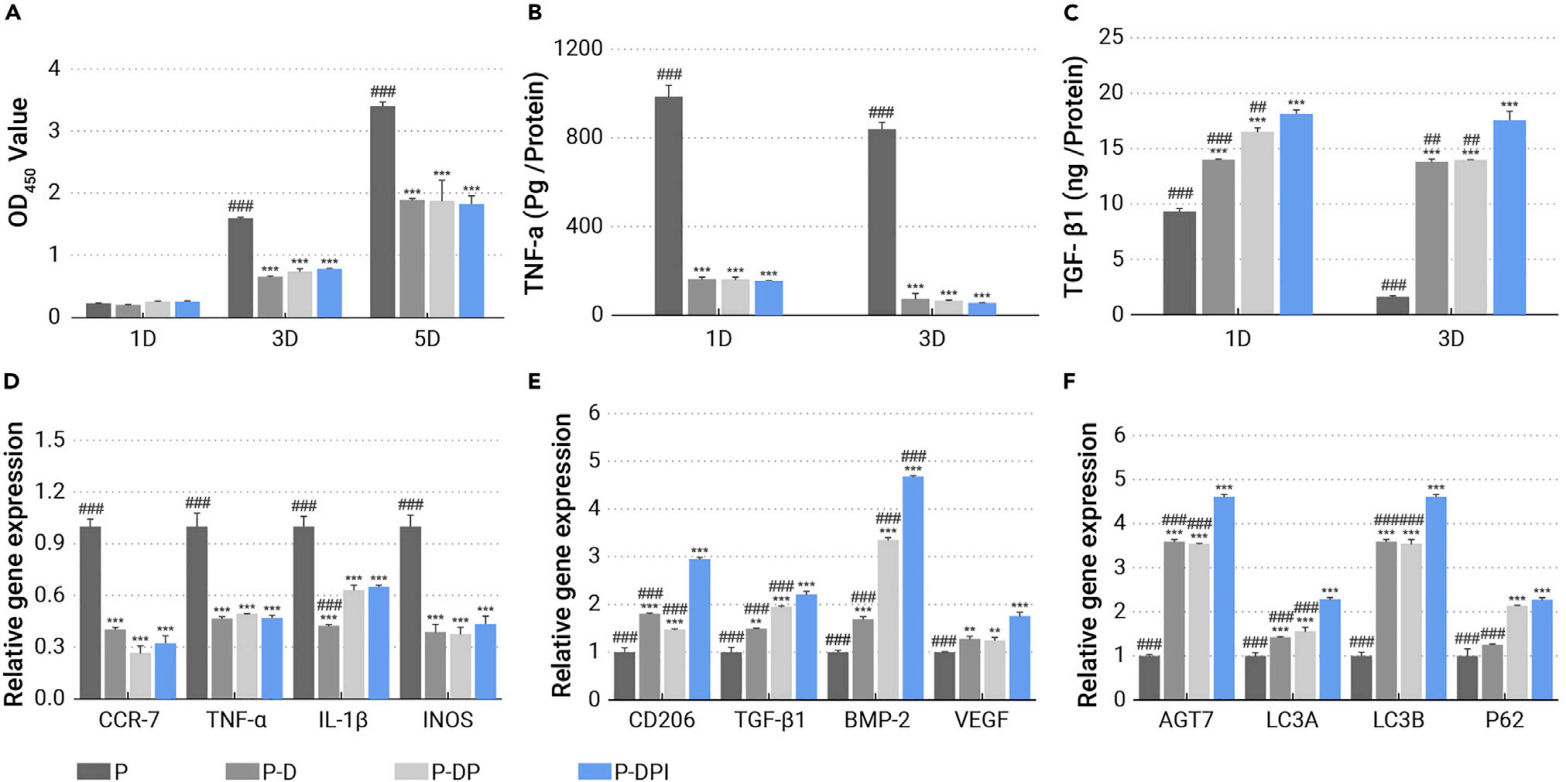
Biological response of macrophages stimulated by various samples (A) Macrophages viability test for 1, 3, and 5 days. (B and C) Secretion of (B) pro-inflammatory and (C) anti-inflammatory cytokines after cultivating macrophages for 1 and 3 days. Expression of (D) M1 and (E) M2 genes, and (F) autophagy-related genes after cultivating macrophages for 3 days. ∗∗p < 0.01 and ∗∗∗p < 0.001 compared with the P group, whereas ##p < 0.01 and ###p < 0.001 compared with the P-DPI group (n = 4).

The immune system is the first line of defense for exogenetic implants and the positive response of immune cells is crucial to bone regeneration.^31^^,^^32^ Here, the states of macrophages cultured on various samples were evaluated on the cellular, protein, and gene levels. The untreated PEEK (P) holds the largest amount of macrophages, whereas addition of the PTMC/DEX coating (P-D), PIII (P-DP), and grafting of IL-10 (P-DPI) slow proliferation (Figure 3A). The viability of macrophages in the P-D, P-DP, and P-DPI groups is less than one-third of that in the P group on the fifth day, indicating the mitigated inflammation at early stage. The macrophages and substrates also show different morphological changes with cultivation time (Figure S4, supplemental information). Adhesion and proliferation of macrophages on the bare PEEK are robust so that the sample surface is covered completely with layers of cells with some cells being unrecognizable individually. In contrast, few macrophages were observed from the modified samples consistent with the CCK-8 results. The early immunological states of macrophages were evaluated by quantitatively detecting the pro-inflammatory tumor necrosis factor alpha (TNF-α) and anti-inflammatory transforming growth factor β 1 (TGF-β1) cytokines after culturing the cells on various samples for 1 and 3 days. The concentration of TNF-α was as high as 800 pg mL^−1^ for the P group, but macrophages on the modified samples secreted much less TNF-α, with the least detected from the P-DPI group (Figure 3B). With regard to anti-inflammatory cytokines, the trend was reversed as the P-DPI group shows the highest concentration of TGF-β1 followed by the P-DP and P-D groups, with the least TGF-β1 detected from the P group (Figure 3C). The results elucidate that the programmed surface stimulates macrophages to secrete more anti-inflammatory cytokines, prohibiting the secretion of pro-inflammatory cytokines, giving rise to less inflammation, which may facilitate the subsequent osteogenesis.^33^^,^^34^

In a next step, the cells cultured in different groups for 3 days were determined by flow cytometry when using chemokine receptor 7 (CCR7) and cluster of differentiation 206 (CD206) as the biomarkers to identify M1 and M2 macrophages, respectively. The percentage of CCR7-positive macrophages was 69.5% in the P group, which decreased to 28.8% in the P-DPI group (Figure S5, supplemental information). On the contrary, the percentage of macrophages tagged with CD206 was increased from 34.1% in the P group to 87.2% in the P-DPI group, indicating significant M2 polarization of macrophages in the latter group. This polarization trend was also determined on the gene level by real-time PCR. Compared with the P group, all the pro-inflammatory genes, including CCR7, TNF-α, IL-1β, and inducible nitric oxide synthase (iNOS), are downregulated (Figures 3D and S6A, supplemental information), but the anti-inflammatory genes, such as CD206, TGF-β1, vascular endothelial growth factor, and bone morphogenetic protein 2 were upregulated (Figures 3E and S6B, supplemental information) in the P-D, P-DP, and P-DPI groups, corroborating the M1 to M2 polarization. As autophagy can stabilize the immune state by demolishing the overloaded inflammation motivators,^35^ the autophagy-related genes of the cultured macrophages are also evaluated by real-time PCR. Figures 3F and S6C (supplemental information) show that all the autophagy-related genes, including autophagy-related 7 protein (ATG7), autophagy-related protein LC3A (LC3A), autophagy-related protein LC3B (LC3B), and sequestosome-1 (P62) of the cells cultured on the modified samples were upregulated within 3 days. Therefore, the autophagy process is reactive and stimulates antigen presentation, in turn enhancing polarization of macrophages from M1 to M2.

### Osteogenesis *in vitro*

**Figure 4:**
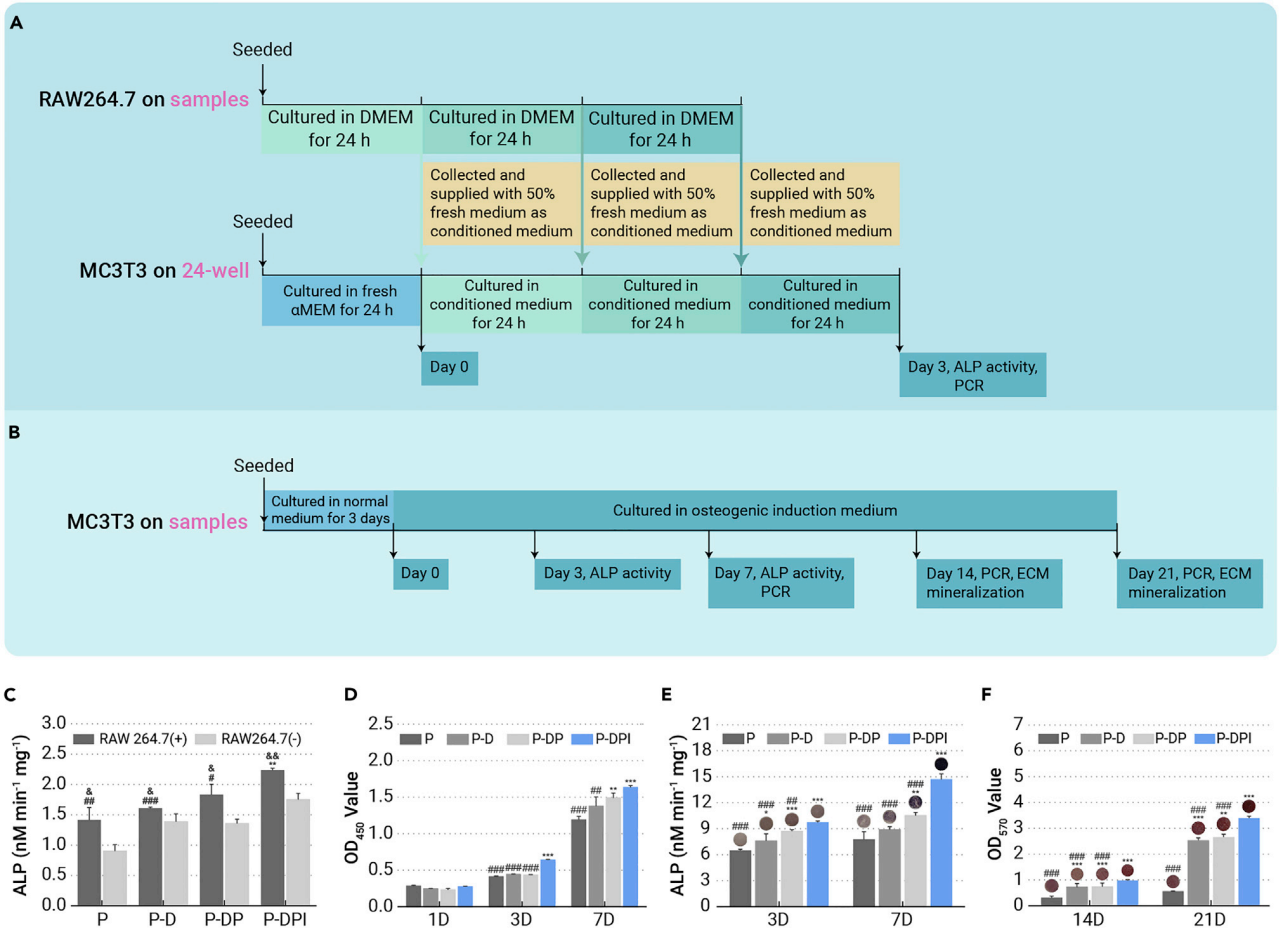
*In vitro* performances of osteoblasts (A) Experimental design of conditioned culture and analysis. (B) Experimental design of direct culture and analysis. (C) ALP activity of osteoblasts in different groups after conditioned culture. (D) Viability of osteoblasts directly cultured on different samples for 1, 3, and 7 days. (E) ALP activity of osteoblasts directly cultured on different samples after osteogenic induction for 3 and 7 days. (F) Mineralization of osteoblasts directly cultured on different samples after osteogenic induction for 14 and 21 days. ∗∗p < 0.01 and ∗∗∗p < 0.001 compared with the P group, #p < 0.05, ##p < 0.01, and ###p < 0.001 compared with the P-DPI group, whereas &p < 0.05, &&p < 0.01, and &&&p < 0.001 by comparing RAW 264.7 (+) with RAW 264.7 (−) in each group (n = 4).

To evaluate whether the immunological response fosters bone formation, the conditioned medium containing cytokines of macrophages treated differently was used to culture osteoblasts (MC3T3-E1 cells) for 3 days according to the experimental design illustrated in Figure 4A. MC3T3-E1 cells respond to the conditioned medium of the modified groups with increased alkaline phosphatase (ALP) activity (Figure 4C). This osteogenic direction was corroborated by the upregulated expression of ALP (Figure S7A, supplemental information), osteopontin (OPN) (Figure S7B, supplemental information), and osteocalcin (OCN) (Figure S7C, supplemental information). The conditioned medium of the P-DPI group elevated osteogenic differentiation, again illustrating the synergistic effects of DEX and IL-10 during the early period. This supports the expectation that a positive immunological response directed by the sequentially releasing surface can promote early osteogenesis and that indirect cultivation validates the immune-mediated regulatory capacity of the P-DPI sample in the initial stage.

Subsequently, the osteogenesis of osteoblasts was evaluated by directly cultivating the cells on different samples for up to 3 weeks. The experimental design of osteogenic culture is illustrated in Figure 4B, and before which the MC3T3-E1 cells were cultured on different samples without osteogenic induction for 1, 3, and 7 days to evaluate the biocompatibility. As shown in Figure 4D, osteoblasts thrive more on the P-DPI sample than those on the other samples, indicating the excellent surface biocompatibility rendered by DEX and IL-10. Early osteogenesis is represented as ALP activity after osteogenic induction and the P-DPI group is superior to other groups (Figure 4E). Osteogenic differentiation of the directly cultured osteoblasts was also analyzed in terms of gene expression. Among the modified groups, the expressions of ALP (Figure S8A, supplemental information), OPN (Figure S8B, supplemental information), and OCN (Figure S8C, supplemental information) at all time points were upregulated, with the P-DPI group faring the best. The improved osteogenic differentiation was further verified by the best mineralization status of cells in the P-DPI group (Figure 4F). The results collectively reveal that steady release of DEX followed by IL-10 further enhances the osteogenic effect during the later weeks. All in all, IL-10 and DEX offer synergistic effects to produce the suitable immunological environment for bone formation. Both immune-mediated regulation and direct osteogenic promotion are crucial to the osseointegration of bone implants, and the sequential release of IL-10 and DEX designed in the P-DPI group can well match the bio-progress of bone formation.

### Inflammation *in vivo*

**Figure 5:**
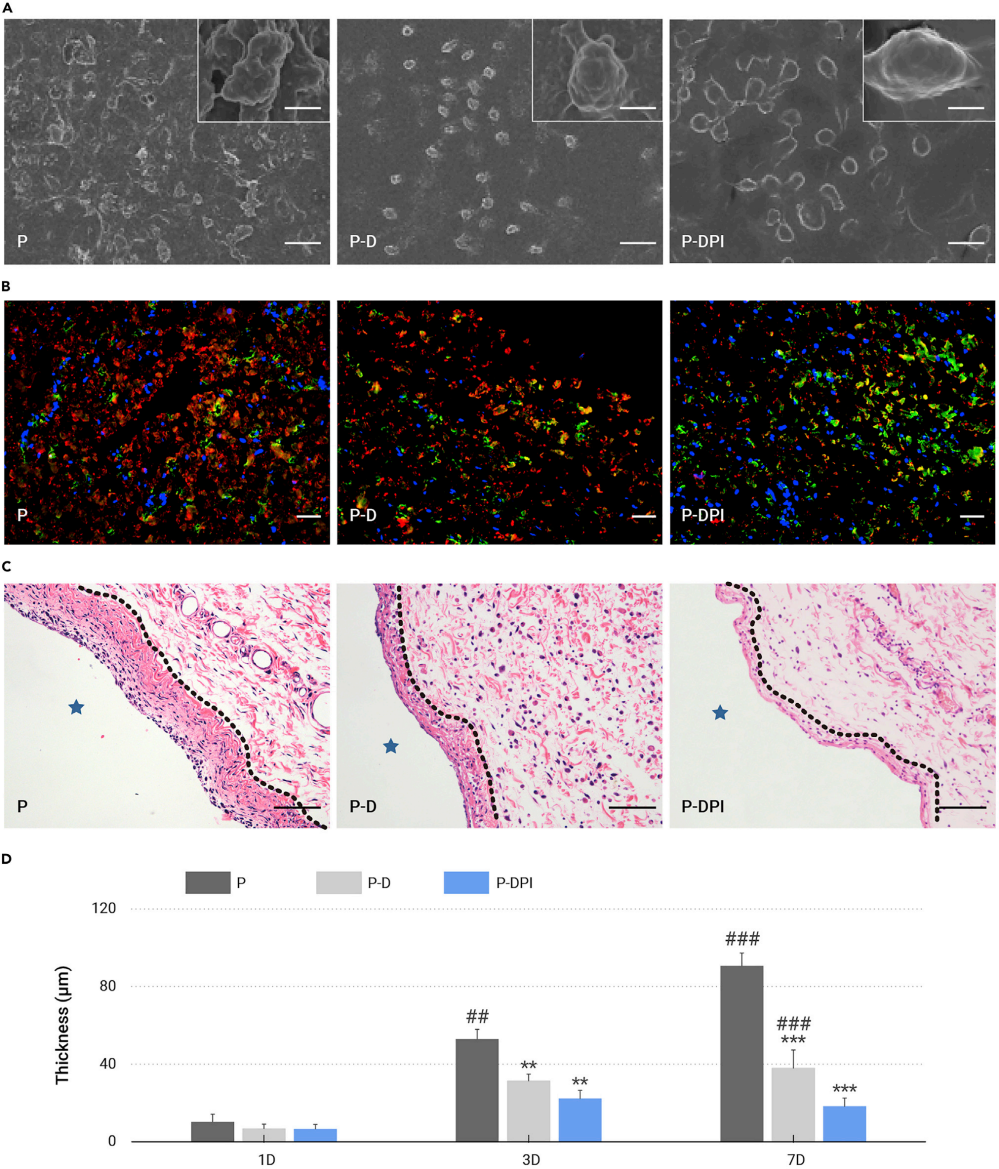
*In vivo* analysis of the inflammatory status (A) SEM images of macrophages on different samples after implantation for 7 days. (B) Immunofluorescent staining images of macrophages on different samples after implantation for 7 days. Red, green, and blue fluorescence reflect density for iNOS, CD163, and nuclei. (C) H&E staining images of peri-implant tissues after implantation for 7 days. The fibrous layers are marked by dashed lines. (D) Quantitative comparison of fibrous layer thickness after implantation for 1, 3, and 7 days. Scale bars, 100 μm (except those in the insets being equal to 10 μm). ∗∗p < 0.01 and ∗∗∗p < 0.001 compared with the P group, whereas ##p < 0.01 and ###p < 0.001 compared with the P-DPI group (n = 6).

*In vivo* inflammatory responses were analyzed from the morphological, immunofluorescent, and histological perspectives. Under SEM observation, the P-D and P-DPI samples showed a slippery surface after implantation for 1 day, but proteins tended to adhere to the bare PEEK, leading to adherence and proliferation of macrophages (red arrows in Figure S9, supplemental information). After implantation for 7 days, the sporadic macrophages in the P-DPI group showed an elongated shape compared with the spherical ones in the other two groups, indicating M2 polarization consistent with the aforementioned *in vitro* results (Figure 5A).^36^ The polarization of macrophages *in vivo* was further determined by immunofluorescent staining of nitric oxide synthase (iNOS as M1 marker) and cluster of differentiation 163 (CD163 as M2 marker). There were many more M2 macrophages detected in the P-DPI group than detected in the P and P-D groups, which validates the positive regulatory effect of P-DPI group (Figures 5B and S10, supplemental information). The inflammatory infiltration peri-implant was observed after hematoxylin and eosin (H&E) staining. The fibrous layer in the P group was the thickest, followed by a thinner layer in the P-D group, and the thinnest in the P-DPI group (Figures 5C and S11, supplemental information). Notably, the thickness of the fibrous layer in the P-DPI group decreased after implantation for 7 days, but those in the other two groups increased gradually (Figure 5D), indicating that DEX and IL-10 work together to relieve inflammation.

### Bone formation *in vivo*

**Figure 6:**
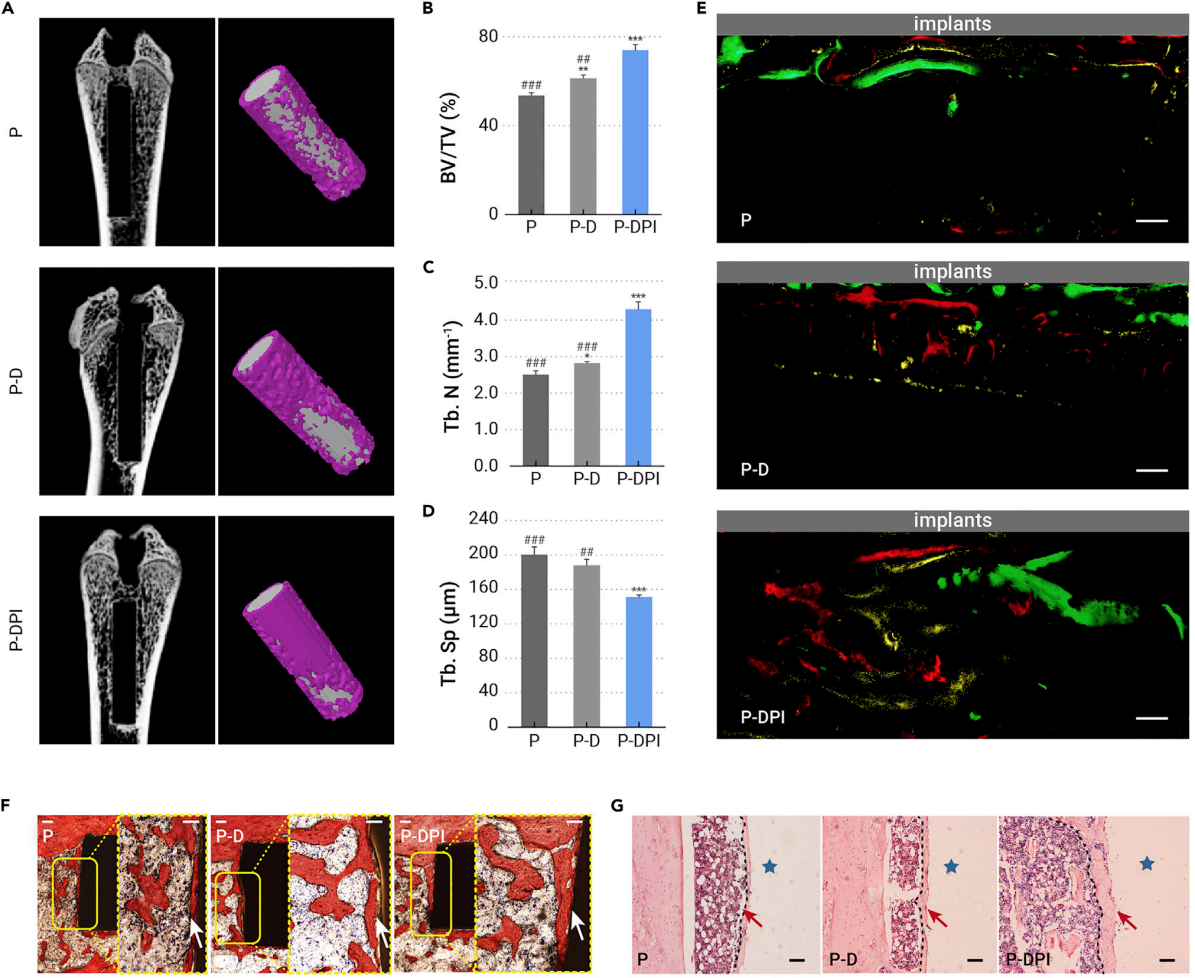
*In vivo* analysis of bone regeneration after implantation for 8 weeks (A) 2D and reconstructed 3D micro-computed tomography (micro-CT) images of the bone with the implants inside. (B–D) Quantitative micro-CT data of (B) bone volume/total volume (BV/TV), (C) trabecular number (Tb.N), and (D) trabecular separation (Tb.Sp). (E) Formation of new bone labeled by sequential fluorescent staining (tissues labeled by alizarin red, tetracycline hydrochloride, and calcein show red, yellow, and green fluorescence, respectively). (F and G) Histological observation of peri-implant tissues after (F) Van Gieson and (G) H&E staining with new bone marked by dashed lines. Scale bars, 100 μm. ∗p < 0.05, ∗∗p < 0.01, and ∗∗∗p < 0.001 compared with the P group, whereas ##p < 0.01 and ###p < 0.001 compared with the P-DPI group (n = 6).

Bone formation is the benchmark determining the *in vivo* osteogenic properties, samples were implanted for up to 8 weeks and evaluated systematically. As shown in Figure 6A, the 2D and reconstructed 3D micro-computed tomography scanning images show that peri-implant bone regeneration in the P-DPI group was much better than that in the P and P-D groups. With a bone volume/total volume of 74%, trabecular number of more than 4 mm^−1^, and trabecular separation of 150 μm (Figures 6B–6D), the P-DPI group possessed the best performance of bone remodeling. The whole osteogenesis process was tracked by sequential fluorescent staining (Figure 6E). Red, yellow, and green fluorescence indicate the new bone formation after implantation for 2, 4, and 6 weeks, respectively, and the total fluorescent area in each group is plotted in Figure S12 (supplemental information). Evidently, the P-DPI group holds the largest fluorescent area, which is 1.8–2.5 times that of the other two groups. Besides the quantity, the quality of new bone was examined from the histological perspective. Van Gieson staining (Figure 6F) and H&E staining (Figure 6G) confirmed that the new bone in the P-DPI group was denser and thicker (white and red arrows, respectively) than that in the P and P-D groups. Notably, the largest ratio of bone-to-implant contact was observed from the P-DPI group (Figure S13, supplemental information). Altogether, the immunological environment created in the first few days and stable DEX release in subsequent weeks led to excellent osteogenesis, as manifested by the quality and quantity of new bone.

Nowadays, the pursuit of implant candidates has delivered PEEK to the site of interest because of the favorable mechanical, as well as chemical stability, and natural radiolucency.^37^, ^38^, ^39^ However, the easily formed fibrous layer on bare PEEK hinders osteointegration, thus raising enthusiasm for various modification strategies.^40^, ^41^, ^42^, ^43^, ^44^, ^45^, ^46^ Previous studies have mainly concentrated on introducing biocompatible elements into the materials and/or constructing functional coatings, the *in vitro* effects of which are plausible but the lack of systematic consideration of immune-mediated regulation has hampered success *in vivo*. Herein, the immune response is taken into full consideration as hinted by recently proposed theories.^47^ Rather than modulating the immune response by adjusting the physical or chemical properties such as the surface roughness/morphology and ion release,^48^^,^^49^ we deliver the synergistic effect of humanized IL-10 and DEX to minimize immune rejection. As an anti-inflammatory cytokine, the cascade of IL-10 in the first couple of days sends a signal that inflammation is relieved and the M1-M2 transition is underway. A positive response from the immune system is then triggered and macrophages secrete more anti-inflammatory cytokines and inhibit production of pro-inflammatory counterparts, with the corresponding genes being regulated in parallel. At the same time, genes related to autophagy are upregulated and, together with the sustained release of anti-inflammatory DEX, reduce inflammation and promote M2 polarization of macrophages. Collectively, the suitable immunological surroundings are created to promote osteogenesis.

This is the first attempt to program PEEK surface to initiate immune mediation and fulfill bone regeneration sequentially. Our results reveal that this strategy is superior to most other approaches in which the functionalized surfaces only play roles in a certain stage after implantation. In our experiments, PEEK is coated with PTMC containing DEX and subjected to N_2_ PIII to facilitate surface grafting of IL-10. The functional molecules are programmed for sequential release that follows the first-order kinetics. Compared with the other biodegradable polymers, PTMC is chosen as the coating material in this study because its degradation via surface erosion is desirable for the sequential release of loaded molecules, and, moreover, the non-acidic products after PTMC degradation contribute to excellent biocompatibility and cause little inflammation.^30^^,^^50^^,^^51^ The cascade of IL-10 and a small amount of DEX creates a suitable immunological environment within 1 week after implantation. By taking advantage of the immunomodulatory effects, osteoblasts thrive on the implant and make a steady transition to the bone-formation stage. In the next few weeks, the stable release of DEX guarantees the quantity and quality of new bone and, consequently, cohesion between the implant and new bone is improved resulting in excellent osseointegration.

It should also be mentioned that previous research activities primarily focus on the functionalization of bone repair materials individually, but the strategies may not be applicable to other regenerative biomaterials. In contrast, this work starts with a comprehensive cognition about different phases of bone regeneration, including immune system activation, polarization of macrophages, differentiation of osteoblasts, bone formation, and bone remodeling, as well as the key factors linking the adjacent phases. The programmed surface is designed to produce a smooth transition through the different phases with minimal side effects and this novel and effective concept can be extended to other prosthetic systems.

### Conclusions

A programmed surface was designed and fabricated to achieve immune-mediated osteogenic regulation. In this process, IL-10 and DEX are released sequentially in a specific time window. The cascade of IL-10 and a small amount of DEX in the first few days hinders inflammation and promotes M2 polarization of the surrounding macrophages, creating a suitable immunological environment for bone regeneration. In the ensuing bone-formation stage, steady release of DEX fosters osteogenesis in terms of both quantity and quality. As a result, new bone is formed on the programmed surface via immune-mediated regulation and robust bone-implant osseointegration is obtained. This novel concept and better understanding bode well for success *in vivo* and provide insights into the design of advanced biomedical implants for tissue engineering and immunotherapeutic applications.

## Lead contact website

http://htod.siat.ac.cn/Team/detail.shtml?id=4&cid=11.

## Supplemental information

Document S1. Supplemental experimental procedures, Figures S1–S11, and Table S1

Document S2. Article plus supplemental information
