# Supplementary material for: Programmed surface on poly(aryl-ether-ether-ketone) initiating immune mediation and fulfilling bone regeneration sequentially
Source: Innovation (Camb). 2021 Aug 5;2(3):100148. doi: 10.1016/j.xinn.2021.100148 (PMC8454576; doi:10.1016/j.xinn.2021.100148)
Supplement: Document S1. Supplemental experimental procedures, Figures S1–S11, and Table S1 [file mmc1.pdf]

The Innovation, Volume *2*

Supplemental Information

Programmed surface on poly(aryl-ether-ether-ketone) initiating immune mediation and fulfilling bone regeneration sequentially

Lingxia Xie, Guomin Wang, Yuzheng Wu, Qing Liao, Shi Mo, Xiaoxue Ren, Liping Tong, Wei Zhang, Min Guan, Haobo Pan, Paul K. Chu, and Huaiyu Wang

Supporting Information

Programmed surface on poly(aryl-ether-ether-ketone) initiating immune mediation and fulfilling bone regeneration sequentially

Lingxia Xie,[1,4] Guomin Wang,[2,4] Yuzheng Wu,[1,2] Qing Liao,[1] Shi Mo,[2] Xiaoxue Ren,[1] Liping Tong,[1] Wei Zhang,[3] Min Guan,[1] Haobo Pan,[1] Paul K. Chu,[2] and Huaiyu Wang[1,*]

[1] Center for Human Tissues and Organs Degeneration, Shenzhen Institute of Advanced Technology, Chinese Academy of Sciences, Shenzhen 518055, China

[2] Department of Physics, Department of Materials Science and Engineering, and Department of Biomedical Engineering, City University of Hong Kong, Tat Chee Avenue, Kowloon, Hong Kong, China

[3] Technical Institute of Physics and Chemistry, Chinese Academy of Sciences, Beijing 100190, China

[4] These authors contributed equally

* Correspondence：hy.wang1@siat.ac.cn

Experimental procedure

Preparation and characterization of samples

PEEK was cut into round plates (diameter = 1.5 cm, thickness = 2 mm, *in vitro*) and cylinders (diameter = 1.5 mm, thickness = 7 mm, *in vivo*), respectively. The samples were polished with sandpaper (800, 1200 and 2000 mesh) and treated ultrasonically with acetone, ethanol, and pure water. The coating of PTMC with DEX (5 wt%) was fabricated on PEEK by solvent evaporation. In detail, DEX and PTMC (1:20) was dissolved in dichloromethane and the solution was spread onto the PEEK samples, vacuum dried at room temperature (0.8 Mpa) for 24 hours until a solid film consisting of PTMC and DEX was formed on the surface. In the next step, the samples were subjected to N_2 PIII (pulse frequency = 1000 Hz, duration = 50 μs, and voltage = 2 kV) for 60 minutes. The treated samples were then immersed in IL-10 (40 ng mL^{-1}) for 24 hours for grafting and then treated with phosphate buffered saline (PBS) to remove loose IL-10. Control and experimental groups are presented as PEEK (P), PEEK + PTMC/DEX coating (P-D), PEEK + PTMC/DEX coating + N_2 PIII (P-DP), and PEEK + PTMC/DEX coating + N_2 PIII + IL-10 grafting (P-DPI).

The surface morphology was examined by SEM (ZEISS SUPRA 55, Germany). Surface hydrophilicity was determined by stastic contact angle measurement (Attension Theta Flex, Biolin Scientific, Sweden) using 4 µL of sessile distilled water under ambient conditions. The surface chemical states were determined by XPS (ESCALAB350Xi, Thermo Fiser, USA) with Al K_α radiation referenced to the Ar 2p peak at 242.4 eV. The adhesion of coating on PEEK substrate was studied using a scratch tester (WS-2005, Zhongke Kaihua Technology, China), with the load ranging from 0 N to 20 N at a speed of 3 mm min^{-1} and 50 N min^{-1}.

Releasing kinetics of IL-10 and DEX

To evaluate the release kinetics of IL-10 and DEX, the P-DPI samples with the hybrid coating (n = 4 per group) were incubated in a solution containing lipase and maintained on a

constant temperature oscillator (37 ˚C) for up to 28 days. The samples were harvested after 1, 3, 5, 7, 14, 21 and 28 days, rinsed with PBS, dried in the air, and dissolved in dichloromethane for elution. The concentrations of IL-10 and DEX in dichloromethane were tested by enzyme-linked immuno sorbent assay (ELISA) using a Mouse IL-10 DuoSet ELISA kit (R&D Systems, USA) and UV-visible spectrophotometry (UV-US, TU-1810, Pulse, China), respectively. The release rates were determined by reverse calculation.

Cell cultivation

Murine-derived macrophages (RAW 264.7) and osteoblasts (MC3T3-E1 cells) from the American Type Culture Collection (ATCC) were utilized in our study and culture at 37 °C. The RAW264.7 cells were maintained in the high-glucose dulbecco's minimum essential medium (DMEM, Hyclone, USA) and the MC3T3-E1 cells were cultured in the alpha minimum essential medium (α-MEM, Hyclone, USA). Fetal bovine serum (10%, Gibco, USA) and penicillin/streptomycin (1% v/v, Invitrogen, USA) were added. All the samples were irradiated by UV for half an hour before cell seeding.

Assessment on RAW 264.7 cells

Proliferation. Samples after sterilization were seeded with 2×10^4 RAW 264.7 cells per sample. After incubating for 1, 3 and 5 days, CCK-8 (Beyotime, China) was used to evaluate cell proliferation. Samples with the cells were then harvested, washed with PBS for three times, and dispersed in 400 μL of culture medium containing 10% CCK-8. After incubation for another 1 hour at 37 °C, the medium was mixed thoroughly and the supernatant (200 μL) was collected to test OD450 using a microplate reader (BL340, Biotech, USA).

Morphology. After incubating for 1, 3 and 5 days, the cells were observed by SEM. For preparation, samples were washed with PBS for three times and then immersed in 2.5% glutaraldehyde for fixation. The fixed samples were sequentially treated with ethanol (30%, 40%, 50%, 70%, 90% and 100% v/v) for dehydration, dried in air, and sputtered with platinum.

Inflammatory factors. After incubating for 1 and 3 days, the expressions of TNF-α and TGF-β1 were examined by ELISA. Supernatant was centrifuged at 2000 × g for 5 minutes and the mouse TGF-β1 Valukine ELISA Kit (R&D, USA) and mouse TNF-α Valukine ELISA Kit (R&D, USA) were used following the manufacturer's instruction. In each assay, the concentration of inflammatory factors was determined refering to the standard curve, the total cell protein in each group is measured by using a bicinchoninic acid protein assay kit (Beyotime, China) for normalization.

Macrophages phenotypes. The phenotypes of macrophages were determined by detecting CCR7 (M1 marker) and CD206 (M2 marker) on cell membrane, respectively. In particular, the cells were trypsinized after being cultured on various samples for 3 days, centrifuged at 1500 r/min for 5 minutes, and rinsed with PBS. The harvested cells were treated with CCR7 (R&D Systems, USA) and CD206 (R&D Systems, USA) antibodies for 30 minutes at 4 ˚C, tested by flow cytometry (Beckman CytoFLEXS, USA).

In a next step, the phenotypes of macrophages were detected in terms of gene expression. After cultivating on various samples for 3 days, culture medium was collected and centrifuged at 1500 r/min (5 min) before the supernatants were collected as the conditioned media for the following experiments. The RNA in the cultured cells was extracted by trizol reagent (Qiagen, Hilden, Germany) and reversely transcribed into cDNA (RevertAid First Strand cDNA Synthesis Kit, Thermo Fisher). The expressions of CCR7, TNF-α, IL1-β and INOS for M1 phenotype and CD206, TGF-β1, BMP-2 and VEGF for M2 phenotype were analyzed by RT-PCR (Bio-Rad CFX 96, Transgen Biotech, China). The forward and reverse primers were shown in Table S1. Furthermore, the gene expressions related to autophagy including ATG7, LC3A,LC3B and P62 were quantitatively determined by RT-PCR as described above.

Osteogenesis of MC3T3-El cells cultivated in conditioned medium

To investigate whether the macrophages could influnce the osteogenesis process, RAW 264.7 cells were firstly cultured on different samples for 1, 2 and 3 days and the medium were collected. Afterwards, the collected medium was mixed with an equal volume of the normal culture medium to compose the conditioned medium for culturing MC3T3-E1 cells. For the RAW 264.7 (-) groups, the different samples without cell seeding were immersed in the medium for up to 3 days, and the medium after immersion was collected to compose the conditioned medium. The MC3T3-E1 cells were cultivated in the normal culture medium for 12 hours before replacing the medium with the conditioned medium. After cultivation for another 3 days, the cells after rinsing were treated with the cell lysis buffer (Beyotime, China). The ALP activity was evaluated by the ALP assay kit (Beyotime, China) and normalized to total intracellular protein amount tested by the bicinchoninic acid protein assay kit (Beyotime, China). The RNA in different groups was subjected to RT-PCR to quantitatively evaluate the osteogenic gene expressions including ALP, OPN and OCN.

Osteogenesis of MC3T3-E1 directly cultivated on samples

After cultavation for 1, 3 and 7 days, proliferation of cells was tested by CCK-8 assay as described above. Furthermore, MC3T3-E1 proliferated on different samples for 3 days was subjected to osteogenic induction by refreshing the culture medium and supplementing with ascorbic acid (50 $\mu g\ ml^{-1}$) and β-glyceryl phosphate (10 mM). 3 and 7 days later, cells on samples were quantitatively analyzed for the ALP activity following the protocols stated above. The ALP was also stained by the BCIP/NBT kit (Beyotime, China) for observation. After induction for 7, 14 and 21 days, gene expression of ALP, OPN and OCN was again evaluated by RT-PCR as described above.

The mineralization state of extracellular matrix was stained by alizarin red (Beyotime, China). After osteogenic induction for 14 and 21 days, samples were taken out, rinsed with PBS for three times, and fixed with 75% ethanol for 1 hour. Afterwards, all the specimens reacted with alizarin red (40 mM, pH = 4.2) for 30 minutes. Distilled water flushing was then

used to remove the unbound stain and bound stain was dissolved by adding 500 μL cetylpyridinium chloride (10%, pH = 7.0). 200 μL of the solution in each group was then used to test OD570 by microplate reader (BL340, Biotech, USA).

Immune-mediated regulation and osteogenesis *in vivo*

3 months old SD rats (male, 200-300g) were maintained under specific pathogen free (SPF) conditions. The animal experiments in this work were approved by the Ethics Committee for Animal Research of Shenzhen Institute of Advanced Technology, Chinese Academy of Sciences.

Immunological assessments after subcutaneous implantation. The experimental animals were anesthetized with 2% sodium pentobarbital (2.3 mL kg^{-1}) before the samples in different groups were implanted subcutaneously. The implantation sites were symmetrically located on both sides of the dorsal midline. The rats were sacrificed after 1, 3 and 7 days and the samples as well as the surrounding tissues were collected, immersed in paraformaldehyde (4%) for fixation and treated with gradient ethanol for dehydration before SEM observation. The M1 to M2 polarization was determined by staining with the corresponding markers (INOS for M1 and CD163 for M2, Servicebio, China). The nuclei of cells were also stained by 4',6-diamidino-2-phenylindole (DAPI, Servicebio, China) before the observation under fluorescent microscopy (Nikon Eclipse CI, Japan). Afterwards, the specimens were embedded with paraffin, sectioned into slices (5 mm in thickness), and subjected to H&E staining to visualize the tissues surrounding the implants.

Osteogenic assessments. The rats were anesthetized with 2% sodium pentobarbital (2.3 mL kg^{-1}) before defects in cylindrical shape were established at the intercondylar notch of the distal femur. The samples in different groups were implanted into the defects and the wounds were carefully sutured. To monitor the peri-implant new bone formation and mineralization, 3 different fluorochromes including alizarin red (30 mg kg^{-1}, Sigma-Aldrich, USA), tetracycline hydrochloride (25 mg kg^{-1}, Sigma-Aldrich, USA), and calcein (20 mg kg^{-1}, Sigma-Aldrich,

USA) were intraperitoneally administered into the rats after implantation for 2, 4 and 6 weeks, respectively. All the rats were sacrificed 8 weeks later. The femurs with implants were harvested and fixed with 4% paraformaldehyde. Micro-CT (SkyScan 1176, Bruker, Germany) was employed to image the newly formed bone with the 3D images reconstructed (Ctvol, Skyscan). By 3D bone morphometric analysis, BV/TV, Tb.N and Tb.Sp in different groups were determined.

After micro-CT scanning, the fixed samples were dehydrated, embedded, sectioned and ground to a thickness of about 50 μm before the fluorescent observation under confocal laser microscopy (TCS SP8, Leica, Germany). The excitation/emission wavelengths were set at 543/620, 405/575, and 488/520 nm for red, yellow and green fluorescence, respectively. The sections were then subjected to VG staining and H&E staining for the observation of bone-implant interfaces.

Statistical analysis

The experiments in this work were performed in triplicate at leastwith the data being shown as mean ± standard deviation. Statistical evaluation was performed using SPSS software, and differences among groups were determined by analysis of variance (ANOVA) with Bonferroni test. Significant difference was defined at $p < 0.05$ and highly significant difference was defined at $p < 0.01$ or $p < 0.001$.

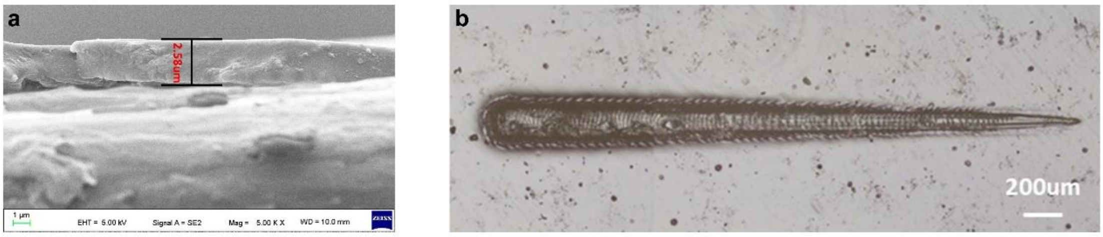

Figure S1: (a) The thickness and (b) scratch test of fabricated coating.

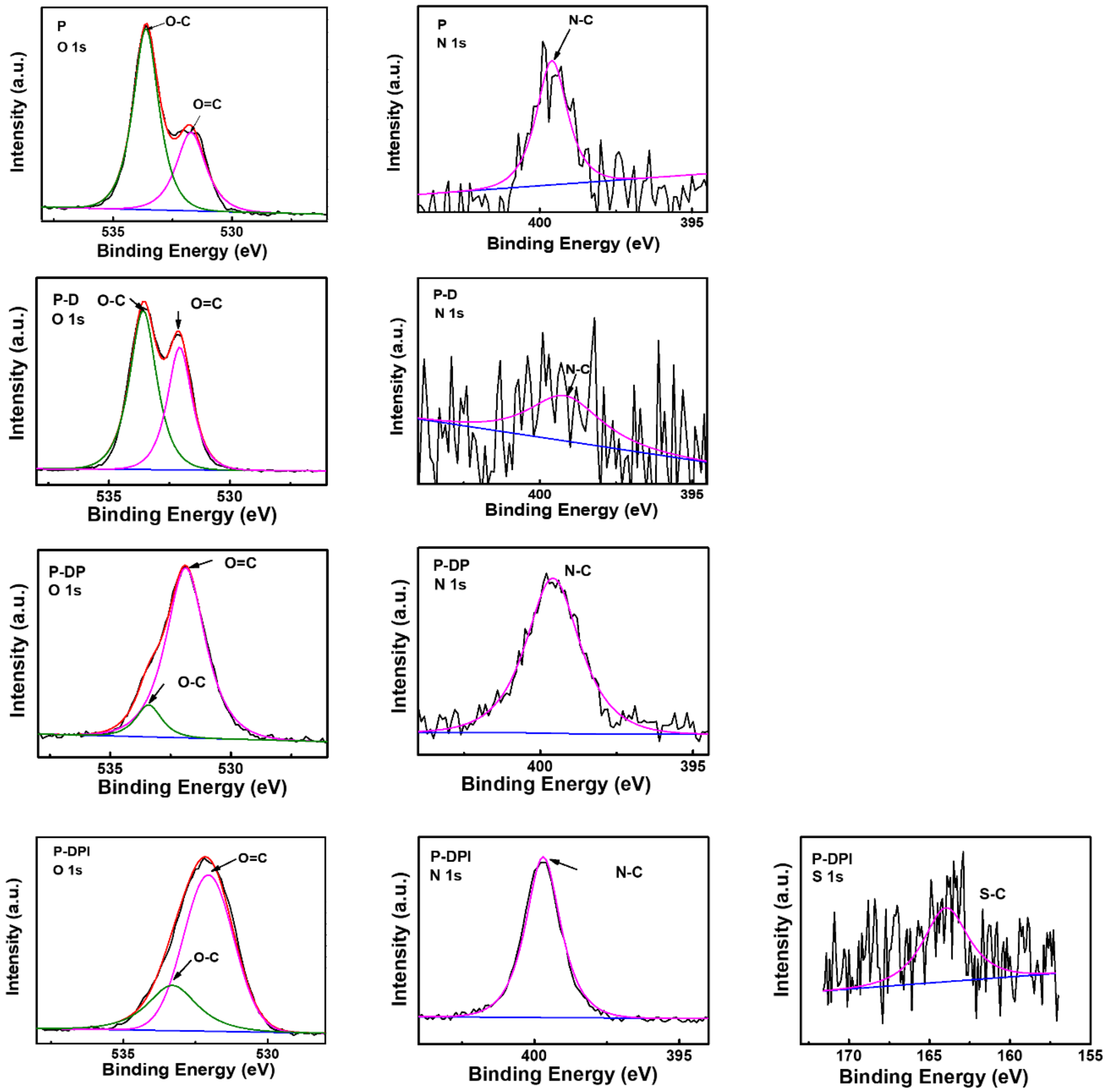

Figure S2: High-resolution XPS spectra for O 1s, N 1s and S 1s acquired from different samples.

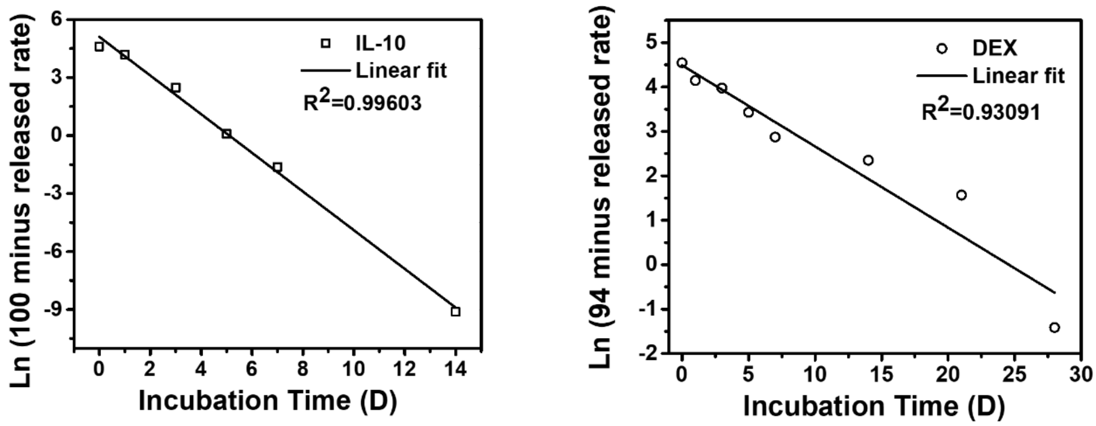

Figure S3: Fitted lines illustrating the relationship between the released rate of IL-10 and DEX on semi-log scale and incubation time.

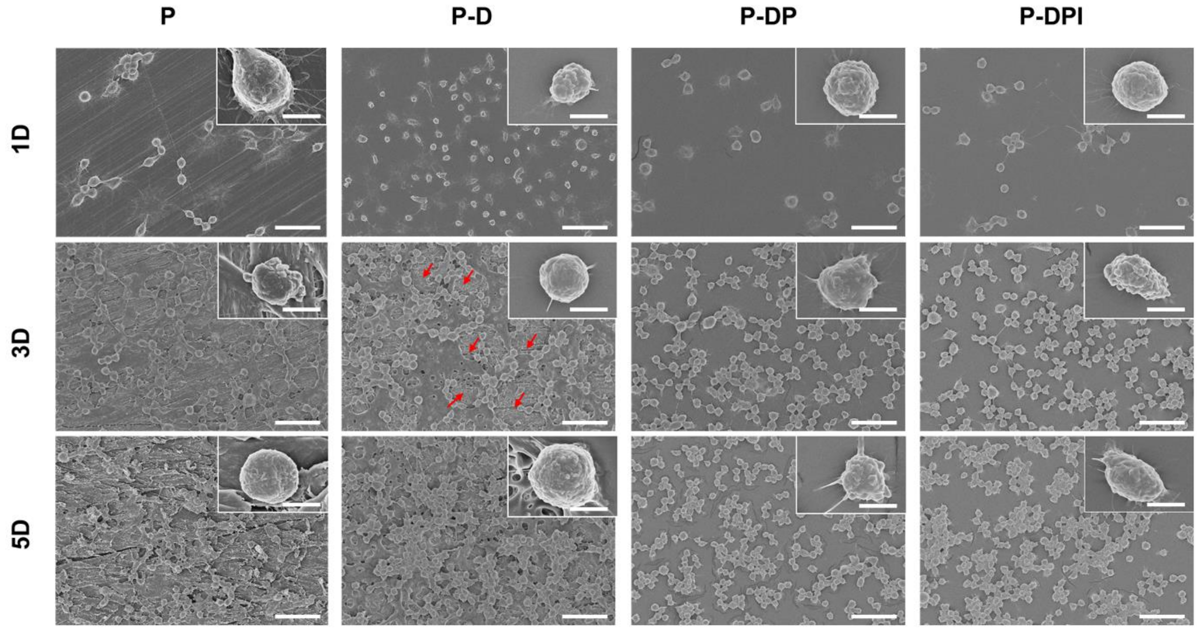

Figure S4: SEM images (scale bar = 35 µm) of macrophages cultivated on samples for 1, 3 and 5 days with insets showing the magnified images (scale bar = 5 µm).

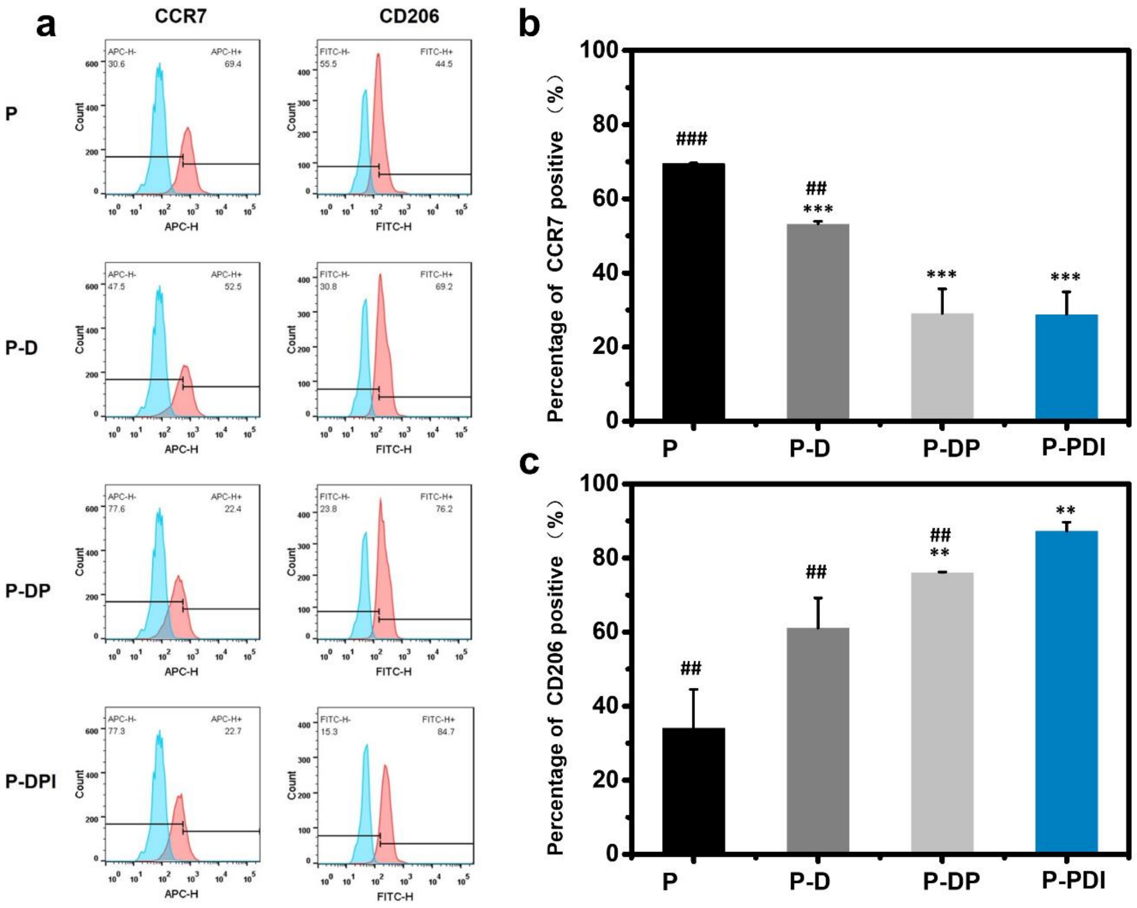

Figure S5: (a) Flow cytometric analysis of RAW264.7 cells cultivated on samples for 3 days; (b) Percentage of CCR7-positive macrophages (M1 phenotype); (c) Percentage of CD206-positive macrophages (M2 phenotype). ** denotes $p < 0.01$ and *** denotes $p < 0.001$ compared with the P group, whereas ## denotes $p < 0.01$ and ### denotes $p < 0.001$ compared with the P-DPI group (n=4).

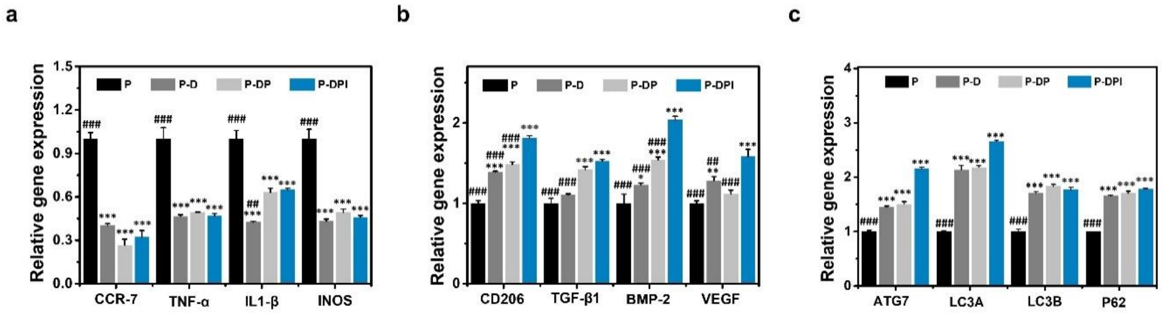

Figure S6: Expression of (a) M1 and (b) M2 genes, and (c) Autophagy-related genes after cultivating macrophages on different samples for 1 day. * denotes $p < 0.01$** denotes $p < 0.01$

and *** denotes $p < 0.001$ compared with the P group, whereas ## denotes $p < 0.01$ and ### denotes $p < 0.001$ compared with the P-DPI group (n=4).

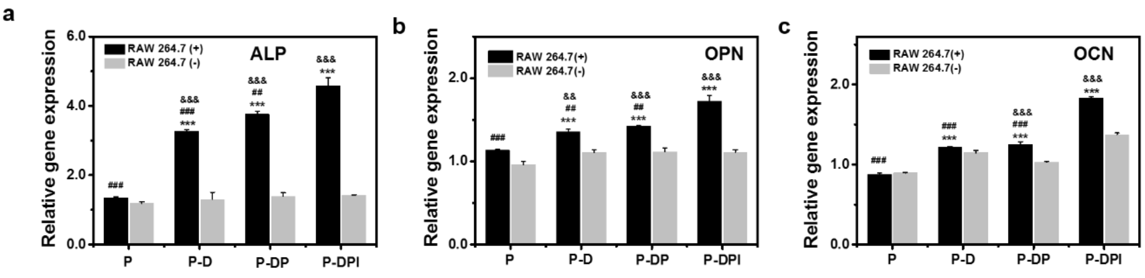

Figure S7: Osteogenic gene expressions of osteoblasts in different groups after conditioned culture: (a) ALP, (b) OPN and (c) OCN. *** denotes $p < 0.001$ compared with the P group, ## denotes $p < 0.01$ and ### denotes $p < 0.001$ compared with the P-DPI group, whereas && denotes $p < 0.01$ and &&& denotes $p < 0.001$ by comparing RAW 264.7 (+) with RAW 264.7 (-) in each group (n=4).

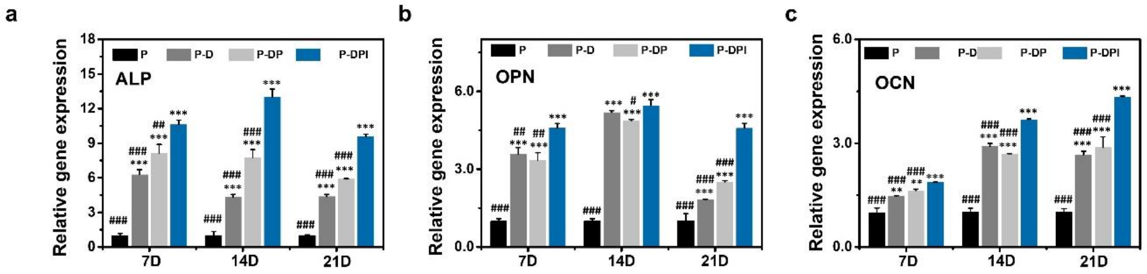

Figure S8: Osteogenic gene expressions of osteoblasts directly cultured on different samples after osteogenic induction for 7, 14 and 21 days: (a) ALP, (b) OPN and (c) OCN. **denotes $p < 0.01$ and *** denotes $p < 0.001$ compared with the P group, whereas # denotes $p < 0.05$, ## denotes $p < 0.01$ and ### denotes $p < 0.001$ compared with the P-DPI group (n=4).

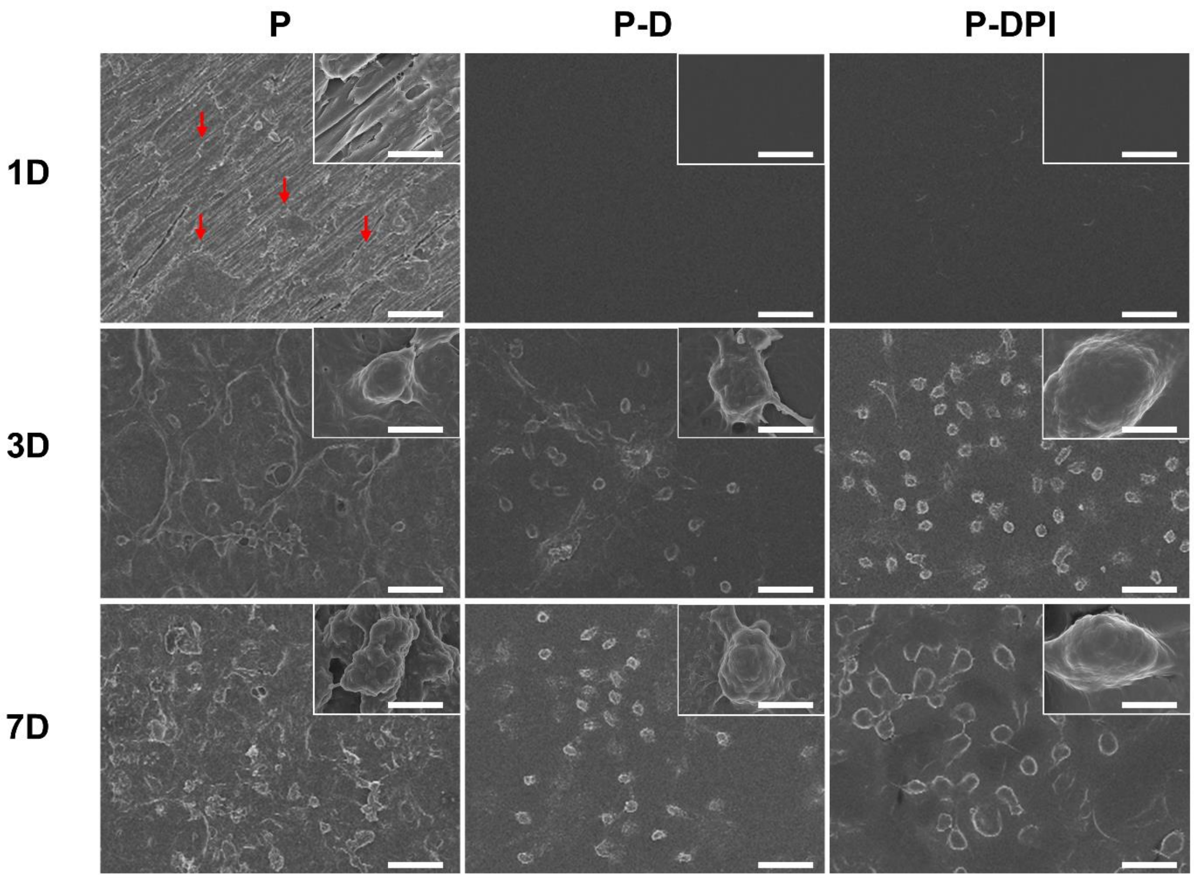

Figure S9: SEM images of macrophages on samples after implantation for 1, 3 and 7 days with insets showing the magnified images (scale bars equal to 100 µm and 10 µm, respectively).

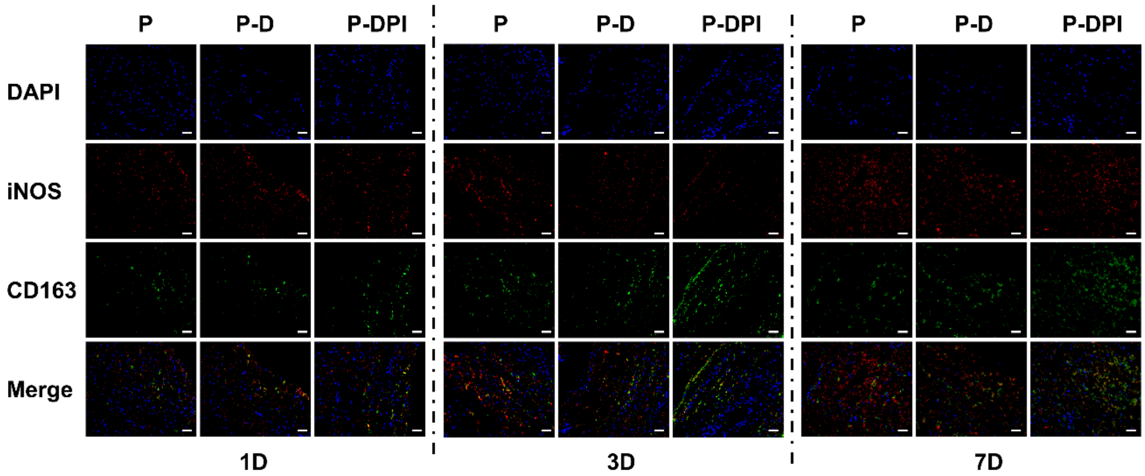

Figure S10: Immunofluorescent staining images of macrophages after implantation for 1, 3 and 7 days: red (INOS for M1), green (CD163 for M2) and blue (nuclei) (scale bar = 100 µm).

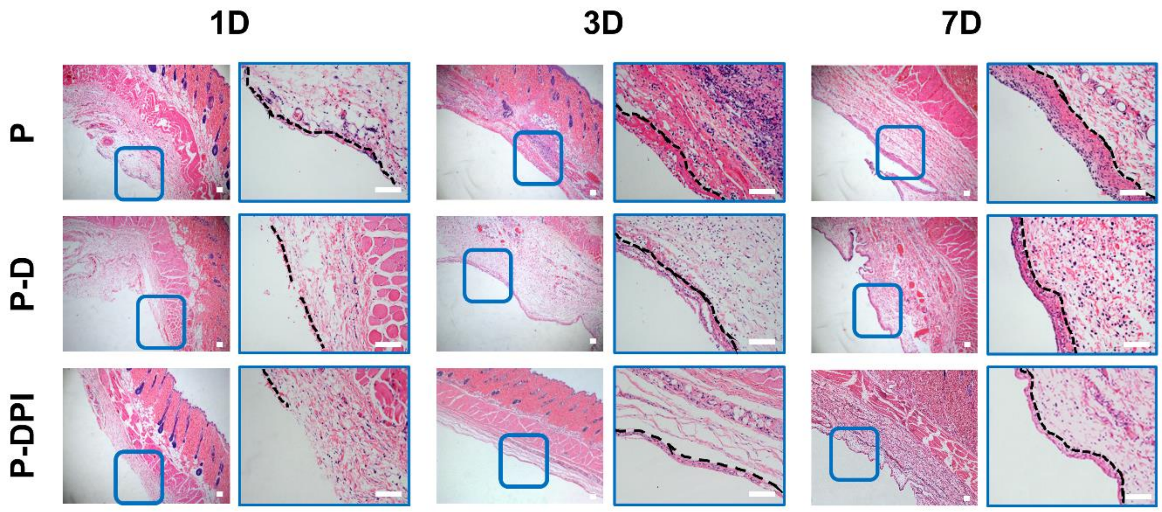

Figure S11: H&E staining images of peri-implant tissues after implantation for 1, 3 and 7 days. The fibrous layers are highlighted by dashed lines (scale bar = 100 μm).

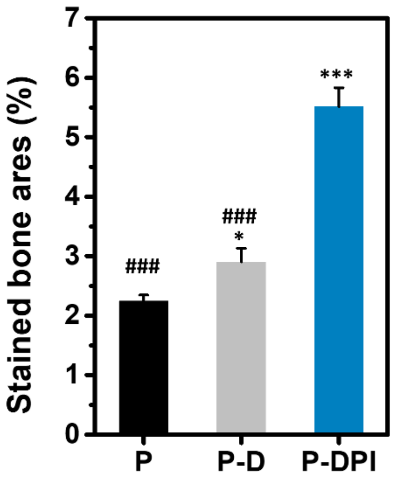

Figure S12: Percentages of stained bone areas in different groups after implantation for 8 weeks. * denotes p <0.05 and *** denotes p <0.001 compared with the P group, whereas ### denotes p < 0.001 compared with the P-DPI group (n=6).

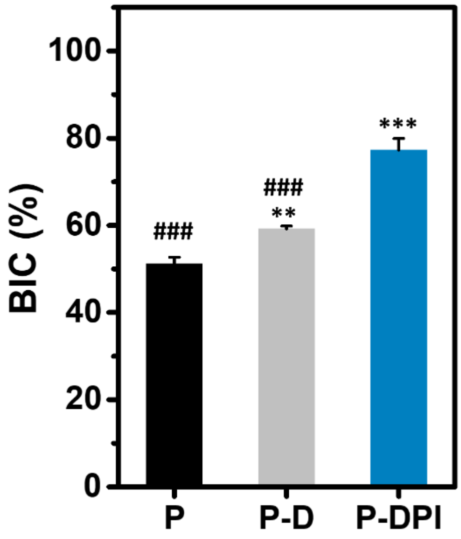

Figure S13: Ratios of BIC in different groups after implantation for 8 weeks. ** denotes p <0.01 and *** denotes p <0.001 compared with the P group, whereas ### denotes p < 0.001 compared with the P-DPI group (n=6).

Table S1. Primers used in RT-PCR.

Primers	Sequences (5'-3')
β-actin	Forward:CGTAAAGACCTCTATGCCAACA
	Reverse: AGCCACCAATCCACACAGAG
CCR7	Forward: GGTGGCTCTCCTTGTCATTTTC
	Reverse: AGGTTGAGCAGGTAGGTATCCG
TNF-α	Forward: TAGCCCACGTCGTAGCAAAC
	Reverse: TGTCTTTGAGATCCATGCCGT
IL-1β	Forward:TGCCACCTTTTGACAGTGATG
	Reverse: GAAGGTCCACGGGAAAGACA
INOS	Forward: GGTGAAGGGACTGAGCTGTTA
	Reverse: TGAAGAGAAACTTCCAGGGGC
CD206	Forward:GCACTGGGTTGCATTGGTTT

	Reverse: CCTGAGTGGCTTACGTGGTT
TGF-β1	Forward:CAGTACAGCAAGGTCCTTGC
	Reverse: ACGTAGTAGACGATGGGCAG
BMP-2	Forward:GCACTGGGTTGCATTGGTTT
	Forward:GGGAAGCAGCAACACTAGAAGA
VEGF	Forward:GTCCCATGAAGTGATCAAGTTC
	Reverse:TCTGCATGGTGATGTTGCTCTCTG
ATG7	Forward:AGCCTGTTCACCCAAAGTTC
	Reverse: CATGTCCCAGATCTCAGCAG
LC3A	Forward:ACAGCATGGTGAGCGTCTC
	Reverse: AGGTTTCTTGGGAGGCGTAG
LC3B	Forward:GATAATCAGACGGCGCTTGC
	Reverse: TCTCACTCTCGTACACTTCGG
P62	Forward:AGCTGCTCTTCGGAAGTCAG
	Reverse: CTCCATCTGTTCCTCTGGCTG
ALP	Forward: TCAGAAGCTAACACCAACG
	Reverse: TTGTACGTCTTGGAGAGGGC
OPN	Forward:TCACCTGTGCCATACCAGTTAA
	Reverse: TGAGATGGGTCAGGGTTTAGC
OCN	Forward: GCAAAGGTGCAGCCTTTGTG
	Reverse: GGCTCCCAGCCATTGATACAG
