# Supplementary material for: Programmed surface on poly(aryl-ether-ether-ketone) initiating immune mediation and fulfilling bone regeneration sequentially
Source: Innovation (Camb). 2021 Aug 5;2(3):100148. doi: 10.1016/j.xinn.2021.100148 (PMC8454576; doi:10.1016/j.xinn.2021.100148)
Supplement: Document S2. Article plus supplemental information [file mmc2.pdf]

Report

Programmed surface on poly(aryl-ether-ether-ketone) initiating immune mediation and fulfilling bone regeneration sequentially

Lingxia Xie,[1,4] Guomin Wang,[2,4] Yuzheng Wu,[1,2] Qing Liao,[1] Shi Mo,[2] Xiaoxue Ren,[1] Liping Tong,[1] Wei Zhang,[3] Min Guan,[1] Haobo Pan,[1] Paul K. Chu,[2] and Huaiyu Wang[1,*]
*Correspondence: hy.wang1@siat.ac.cn
Received: April 27, 2021; Accepted: July 26, 2021; Published Online: August 5, 2021; https://doi.org/10.1016/j.xinn.2021.100148
© 2021 The Author(s). This is an open access article under the CC BY license (http://creativecommons.org/licenses/by/4.0/).

Graphical abstract

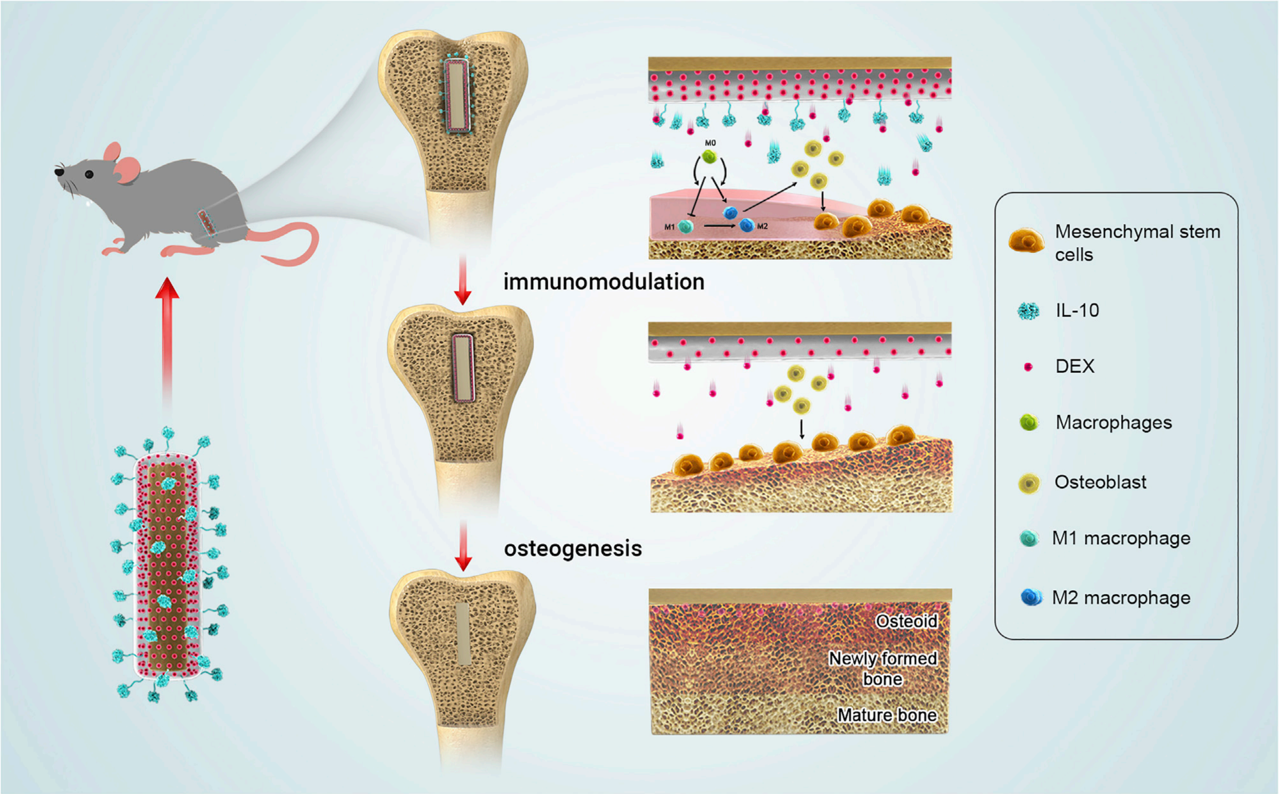

Public summary

- A programed surface is designed and fabricated for immune-mediated osteogenesis
- The degradation of PTMC coating enables a sequential release of IL-10 and DEX
- Initially, osteoimmunomodulation is achieved by IL-10 and a small amount of DEX
- Afterwards, sustained release of DEX fosters the peri-implant bone regeneration

Programmed surface on poly(aryl-ether-ether-ketone) initiating immune mediation and fulfilling bone regeneration sequentially

Lingxia Xie,[1,4] Guomin Wang,[2,4] Yuzheng Wu,[1,2] Qing Liao,[1] Shi Mo,[2] Xiaoxue Ren,[1] Liping Tong,[1] Wei Zhang,[3] Min Guan,[1] Haobo Pan,[1] Paul K. Chu,[2] and Huaiyu Wang[1,*]

[1]Center for Human Tissues and Organs Degeneration, Shenzhen Institute of Advanced Technology, Chinese Academy of Sciences, Shenzhen 518055, China

[2]Department of Physics, Department of Materials Science and Engineering, and Department of Biomedical Engineering, City University of Hong Kong, Tat Chee Avenue, Kowloon, Hong Kong, China

[3]Technical Institute of Physics and Chemistry, Chinese Academy of Sciences, Beijing 100190, China

[4]These authors contributed equally

*Correspondence: hy.wang1@siat.ac.cn

Received: April 27, 2021; Accepted: July 26, 2021; Published Online: August 5, 2021; https://doi.org/10.1016/j.xinn.2021.100148

© 2021 The Author(s). This is an open access article under the CC BY license (http://creativecommons.org/licenses/by/4.0/).

Citation: Xie L., Wang G., Wu Y., et al., (2021). Programmed surface on poly(aryl-ether-ether-ketone) initiating immune mediation and fulfilling bone regeneration sequentially. The Innovation **2(3)**, 100148.

The immune responses are involved in every stage after implantation but the reported immune-regulated materials only work at the beginning without fully considering the different phases of bone healing. Here, poly(aryl-ether-ether-ketone) (PEEK) is coated with a programmed surface, which rapidly releases interleukin-10 (IL-10) in the first week and slowly delivers dexamethasone (DEX) up to 4 weeks. Owing to the synergistic effects of IL-10 and DEX, an aptly weak inflammation is triggered within the first week, followed by significant M2 polarization of macrophages and upregulation of the autophagy-related factors. The suitable immunomodulatory activities pave the way for osteogenesis and the steady release of DEX facilitates bone regeneration thereafter. The sequential immune-mediated process is also validated by an 8-week implementation on a rat model. This is the first attempt to construct implants by taking advantage of both immune-mediated modulation and sequential regulation spanning all bone regeneration phases, which provides insights into the fabrication of advanced biomaterials for tissue engineering and immunological therapeutics.

Keywords: poly(aryl-ether-ether-ketone); surface modifications; sequential release; immune-mediated osteogenesis; bone regeneration

INTRODUCTION

Long-lasting implants that can induce bone remodeling are highly expected to obviate the second surgery arising from unsuccessful bone regeneration.[1–3] However, previous research activities have mainly modified the mechanical and biochemical properties of artificial implants including poly(aryl-ether-ether-ketone) (PEEK), but success *in vitro* may not preclude the excessive inflammation and/or poor bone integration hindering *in vivo* realization.[4–6] The inconsistency between *in vitro* and *in vivo* experiments mainly stems from insufficient consideration of the whole osteogenesis process, which is impacted by multiple factors in the human body.[7–9] Hence, the design of smart bone-implant materials based on an in-depth insight of bone regeneration will work more efficiently than the regulation of individual attributes using a trial-and-error method.[10]

Recently, a better understanding of the bone regeneration process after surgical implantation has been obtained.[11,12] The consensus is that bone regeneration after implantation is a dynamic process which comprises the different phases of inflammation, bone formation, and bone remodeling that are impacted by the surrounding micro-environments.[10,13,14] Within hours after implantation, the immune system is triggered with M1 macrophages secreting inflammation-related mediators and cytokines and small amounts of them are required for bone healing.[15–17] Subsequently, smooth and timely polarization from M1 to M2 enables bone formation with matrix vascularization.[18–20] Both the inflammation and subsequent transformation processes are pivotal but, in most cases, fibrotic capsules induced by the excessive accumulation of inflammatory factors compromise bone-implant osseointegration and increase the risk of implant failure.[20,21] Although some biomaterials have been proposed to accelerate bone formation by interfering with the immune response, they primarily work during the very early stage after implantation but serious immunological rejection may be triggered afterward.[22]

On the molecular level, humanized interleukin-10 (IL-10) is a vital cytokine that helps macrophages adapt to the M2 phenotype and thus limit the inflammatory response *in vivo*.[23–25] Besides, glucocorticoids are inherently anti-inflammatory and the steady and controlled release at a low dose can stimulate bone formation during the early weeks after implantation.[26,27] Inspired by the conceptual molecular understanding of progressive bone formation, a surface co-functionalized with IL-10 and glucocorticoids that can be released orderly with the proper concentration is expected to tune M1-M2 polarization in the early inflammatory stage and promote osteogenetic differentiation thereafter. This can in turn create positive feedback by inhibiting inflammation to consequently foster bone formation. In this way, bone regeneration is accomplished by means of immune-mediated regulation. However, despite the prospect and potential, little effort was devoted to surface functionalization of bone implants that can program the peri-implant response in sequence for yielding the desirable immune-mediated regulation.

In this work, based on a comprehensive understanding of bone-implant interactions, humanized IL-10 and dexamethasone (DEX) (a well-used glucocorticoid) are synergistically utilized to modify PEEK implants to initiate the immunomodulation by cascade of IL-10 and a small amount of released DEX shortly after implantation. Subsequently, steady delivery of DEX during the following weeks allows smooth osteogenesis and bone formation throughout the process. By continuously building new bone with high quantity and quality, excellent bone remodeling can be accomplished *in vivo*. The programmed surface modification strategy that can promote bone regeneration by sequential regulation sheds light on the design of advanced biomedical implants.

RESULTS AND DISCUSSION

Sample characterization

Figure 1A displays a flow chart illustrating the sample preparation process and the as-prepared samples were observed under scanning electron microscopy (SEM). The PEEK sample (defined as P) has a flat surface with minor scratches and, after addition of polytrimethylene carbonate (PTMC) and DEX (defined as P-D), fine dispersion was observed (Figure 1B). The PTMC/DEX coating was not impacted by N_2 plasma immersion ion implantation (N_2

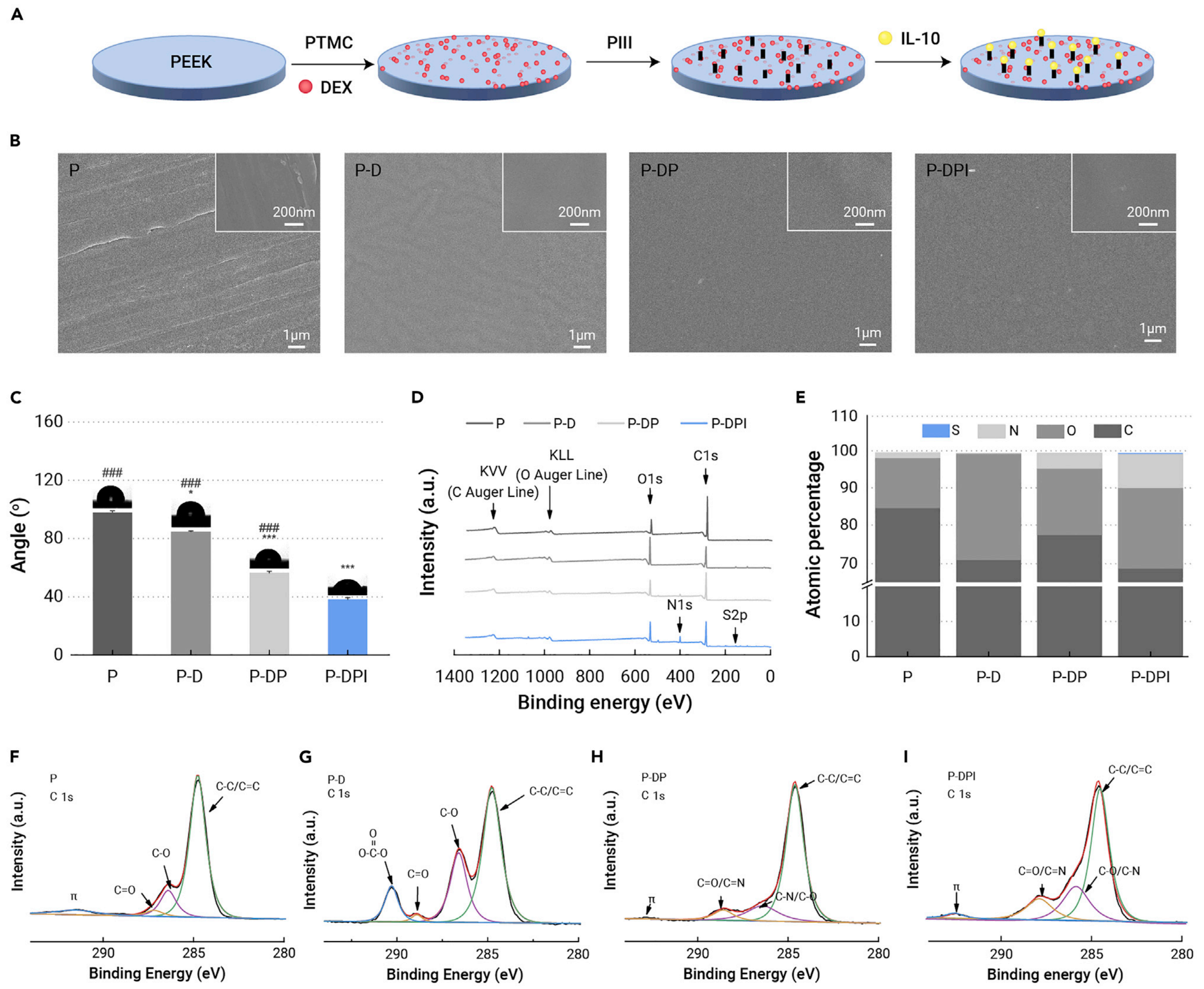

Figure 1. Sample fabrication and characterization (A) Flow chart showing the progress of sample fabrication. (B) SEM images showing the surface morphology of different samples. (C) Water contact angles of samples (n = 4). (D and E) (D) Survey XPS spectra as well as (E) atomic percentages determined on different samples. (F–I) High-resolution C 1s spectra of (F) P, (G) P-D, (H) P-DP, and (I) P-DPI samples. *$p < 0.05$ and ***$p < 0.001$ compared with the P group, whereas ###$p < 0.001$ compared with the P-DPI group.

PIII) (defined as P-DP), and the homogeneous topography after subsequent grafting of IL-10 (defined as P-DPI) suggests that IL-10 is uniformly introduced onto the surface. In addition, the fabricated coating was about 2.58 μm in thickness, which shows a sufficient binding with the PEEK substrate (Figure S1, supplemental information). As shown in Figure 1C, the bare PEEK substrate (P) is hydrophobic but the PTMC/DEX-coated sample (P-D) shows a smaller water contact angle due to the hydrophilic DEX molecules. The surface hydrophilicity of modified samples was further improved by N_2 PIII treatment (P-DP), which on the other hand helps build the cohesion between the substrate and IL-10. Grafting of IL-10 reduces the water contact angle to 38° (P-DPI) and the resulting hydrophilic surface is expected to foster the attachment of osteoblasts.[28]

The chemical states on various samples are examined by X-ray photoelectron spectroscopy (XPS). Peaks for C 1s and O 1s emerged in all samples and a N 1s peak was observed after N_2 PIII treatment (P-DP, Figure 1D). The enhanced N 1s and S 2p peaks detected from the P-DPI sample arise from the grafted IL-10. The effectiveness of surface modification in each step was verified by determining the percentages of different chemical groups (Figure 1E). The high-resolution C 1s spectrum shows C-C/C=C, C-O, and C=O peaks of the P sample (Figure 1F) and the peak at 290.3 eV of the P-D sample is associated with OOC=O in PTMC (Figure 1G). On the P-DP sample, nitrogen bonds with carbon forming C=N and C-N, but C=O/C-O decreases due to energetic plasma bombardment (Figure 1H). After grafting of IL-10, C=N and C-N of the P-DPI sample are more prominent, as shown in Figure 1I. The chemical changes were confirmed by the high-resolution spectra of N 1s, O 1s, and S 1s, as shown in Figure S2 (supplemental information). The characterization results above indicate the effectiveness of surface functionalization of PEEK.

Release kinetics of IL-10 and DEX

A series of tests were carried out to evaluate how biomolecules are released sequentially (Figure 2). With reference to previous studies,[29,30] lipase was added to the solution to mimic the *in vivo* environment that triggers PTMC degradation. As shown in Figure 2A, a rapid release of IL-10 was detected from the P-DPI sample during the initial 5 days and more than 15 ng of IL-10 were released to the solution by the fifth day, constituting more than 90% of the total grafted amount (black symbols). This is also reflected by the release velocity which begins as high as 5 ng day^{-1} but falls precipitously to zero afterward (black line, Figure 2B). About 100% of the grafted IL-10 was released within 7 days, but the release curve of DEX is somewhat different from that of IL-10. In particular, PTMC containing DEX degrades gradually and 110 μg (60%) of DEX were dissolved in the solution

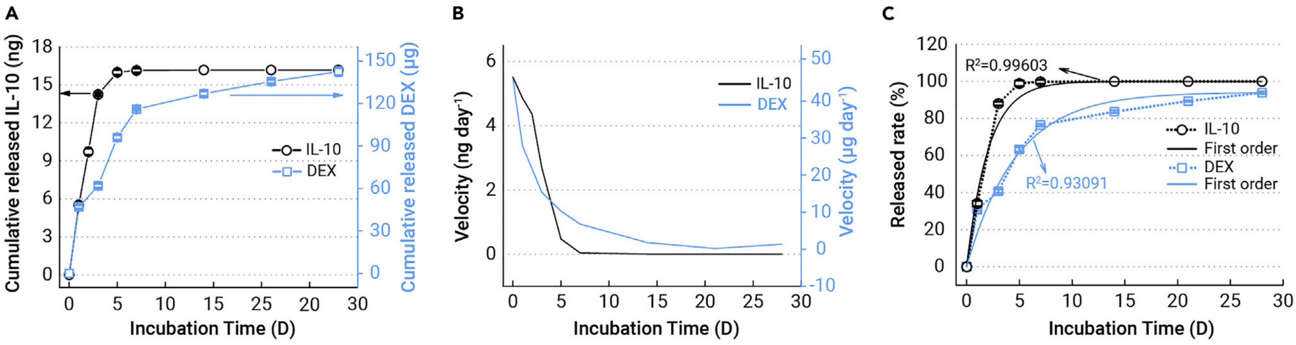

Figure 2. Release parameters of IL-10 and DEX of P-DPI samples in the solution containing lipase as a function of time (A) Cumulative released amounts. (B) Release velocities. (C) Percentages of cumulative release (symbols and dashed lines) as well as curves showing the first-order releasing kinetics (solid lines) (n = 4).

during the first week (blue symbols, Figure 2A). Afterward, the remaining DEX was released at a slower pace and it took more than 4 weeks for the release rate to reach zero (blue line, Figure 2B). The data for these two molecules were fitted with different equations and the release curves obey first-order kinetics with coefficients of 0.99603 for IL-10 and 0.93091 for DEX (Figures 2C and S3, supplemental information). These two release curves are consistent with the intended design that IL-10 grafted on top is for primary control in the first few days and DEX coated with PTMC is maintained at an effective level for the next few weeks. As a result, IL-10 is supposed to trigger the M2 polarization of macrophages in the early stage and DEX can produce continuous effects to promote osteogenesis thereafter.

Immunological response *in vitro*

The immune system is the first line of defense for exogenetic implants and the positive response of immune cells is crucial to bone regeneration.[31,32] Here, the states of macrophages cultured on various samples were evaluated on the cellular, protein, and gene levels. The untreated PEEK (P) holds the largest amount of macrophages, whereas addition of the PTMC/DEX coating (P-D), PIII (P-DP), and grafting of IL-10 (P-DPI) slow proliferation (Figure 3A). The viability of macrophages in the P-D, P-DP, and P-DPI groups is less than one-third of that in the P group on the fifth day, indicating the mitigated inflammation at early stage. The macrophages and substrates also show different morphological changes with cultivation time (Figure S4, supplemental information). Adhesion and proliferation of macrophages on the bare PEEK are robust so that the sample surface is covered completely with layers of cells with some cells being unrecognizable individually. In contrast, few macrophages were observed from the modified samples consistent with the CCK-8 results. The early immunological states of macrophages were evaluated by quantitatively detecting the pro-inflammatory tumor necrosis factor alpha (TNF-α) and anti-inflammatory transforming growth factor β 1 (TGF-β1) cytokines after culturing the cells on various samples for 1 and 3 days. The concentration of TNF-α was as high as 800 pg mL^{-1} for the P group, but macrophages on the modified samples secreted much less TNF-α, with the least detected from the P-DPI group (Figure 3B). With regard to anti-inflammatory cytokines, the trend was reversed as the P-DPI group shows the highest concentration of TGF-β1 followed by the P-DP and P-D groups, with the least TGF-β1 detected from the P group (Figure 3C). The results elucidate that the programmed surface stimulates

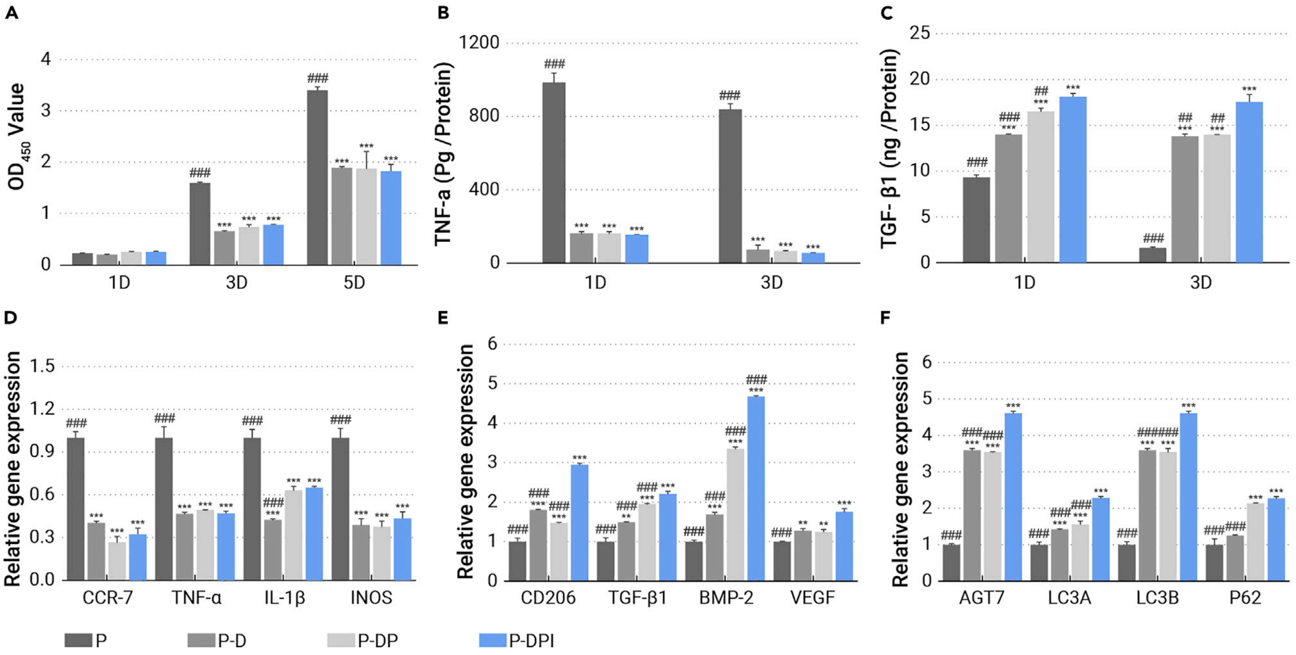

Figure 3. Biological response of macrophages stimulated by various samples (A) Macrophages viability test for 1, 3, and 5 days. (B and C) Secretion of (B) pro-inflammatory and (C) anti-inflammatory cytokines after cultivating macrophages for 1 and 3 days. Expression of (D) M1 and (E) M2 genes, and (F) autophagy-related genes after cultivating macrophages for 3 days. **p < 0.01 and ***p < 0.001 compared with the P group, whereas ##p < 0.01 and ###p < 0.001 compared with the P-DPI group (n = 4).

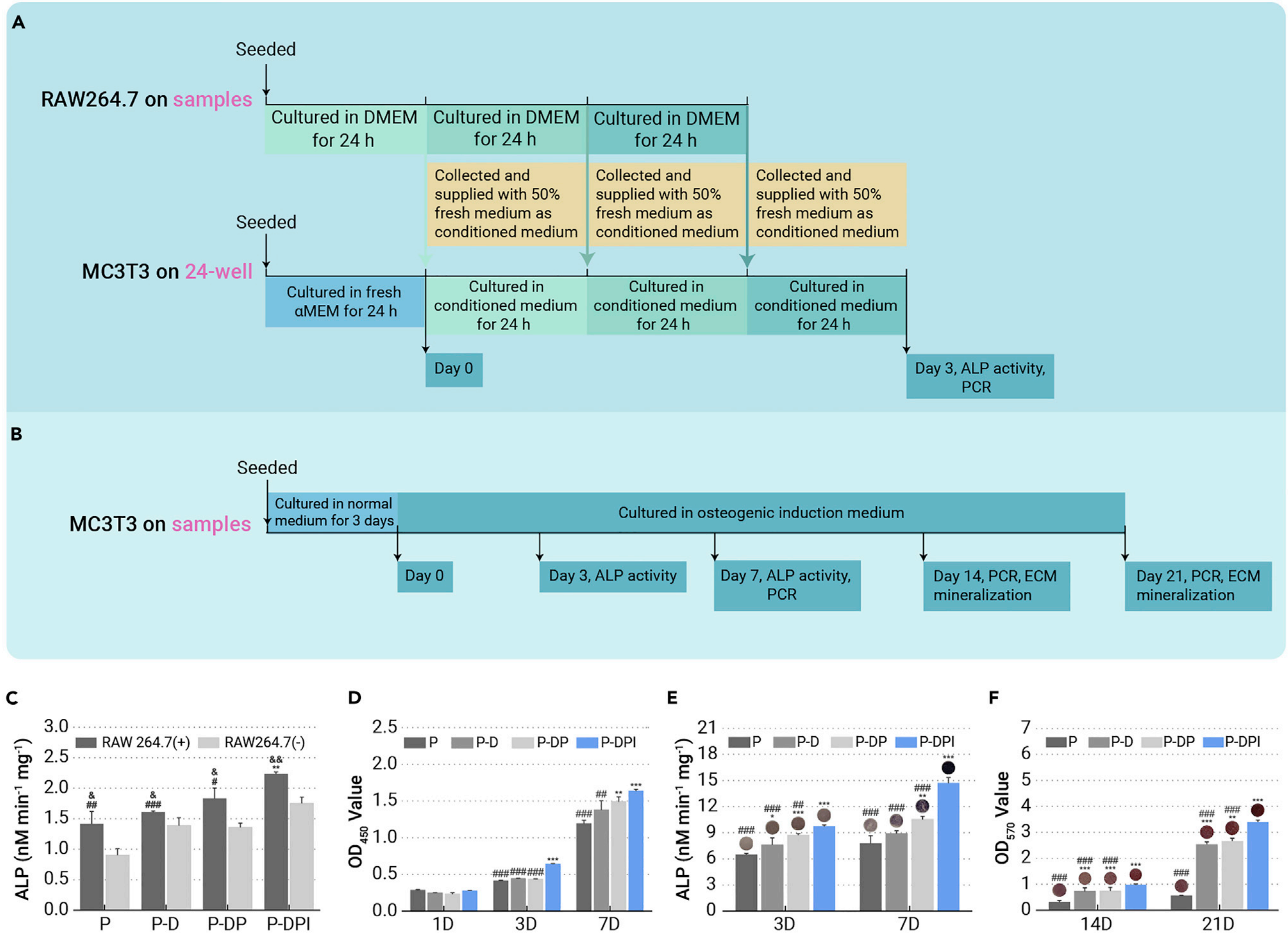

Figure 4. *In vitro* performances of osteoblasts (A) Experimental design of conditioned culture and analysis. (B) Experimental design of direct culture and analysis. (C) ALP activity of osteoblasts in different groups after conditioned culture. (D) Viability of osteoblasts directly cultured on different samples for 1, 3, and 7 days. (E) ALP activity of osteoblasts directly cultured on different samples after osteogenic induction for 3 and 7 days. (F) Mineralization of osteoblasts directly cultured on different samples after osteogenic induction for 14 and 21 days. **p < 0.01 and ***p < 0.001 compared with the P group, #p < 0.05, ##p < 0.01, and ###p < 0.001 compared with the P-DPI group, whereas &p < 0.05, &&p < 0.01, and &&&p < 0.001 by comparing RAW 264.7 (+) with RAW 264.7 (–) in each group (n = 4).

macrophages to secrete more anti-inflammatory cytokines, prohibiting the secretion of pro-inflammatory cytokines, giving rise to less inflammation, which may facilitate the subsequent osteogenesis.[33,34]

In a next step, the cells cultured in different groups for 3 days were determined by flow cytometry when using chemokine receptor 7 (CCR7) and cluster of differentiation 206 (CD206) as the biomarkers to identify M1 and M2 macrophages, respectively. The percentage of CCR7-positive macrophages was 69.5% in the P group, which decreased to 28.8% in the P-DPI group (Figure S5, supplemental information). On the contrary, the percentage of macrophages tagged with CD206 was increased from 34.1% in the P group to 87.2% in the P-DPI group, indicating significant M2 polarization of macrophages in the latter group. This polarization trend was also determined on the gene level by real-time PCR. Compared with the P group, all the pro-inflammatory genes, including CCR7, TNF-α, IL-1β, and inducible nitric oxide synthase (iNOS), are downregulated (Figures 3D and S6A, supplemental information), but the anti-inflammatory genes, such as CD206, TGF-β1, vascular endothelial growth factor, and bone morphogenetic protein 2 were upregulated (Figures 3E and S6B, supplemental information) in the P-D, P-DP, and P-DPI groups, corroborating the M1 to M2 polarization. As autophagy can stabilize the immune state by demolishing the overloaded inflammation motivators,[35] the autophagy-related genes of the cultured macrophages are also evaluated by real-time PCR. Figures 3F and S6C (supplemental information) show that all the autophagy-related genes, including autophagy-related 7 protein (ATG7), autophagy-related protein LC3A (LC3A), autophagy-related protein LC3B (LC3B), and sequestosome-1 (P62) of the cells cultured on the modified samples were upregulated within 3 days. Therefore, the autophagy process is reactive and stimulates antigen presentation, in turn enhancing polarization of macrophages from M1 to M2.

Osteogenesis *in vitro*

To evaluate whether the immunological response fosters bone formation, the conditioned medium containing cytokines of macrophages treated differently was used to culture osteoblasts (MC3T3-E1 cells) for 3 days according to the experimental design illustrated in Figure 4A. MC3T3-E1 cells respond to the conditioned medium of the modified groups with increased alkaline phosphatase (ALP) activity (Figure 4C). This osteogenic direction was corroborated by the upregulated expression of ALP (Figure S7A, supplemental information), osteopontin (OPN) (Figure S7B, supplemental information), and osteocalcin (OCN) (Figure S7C, supplemental information). The conditioned medium of the P-DPI group elevated osteogenic differentiation, again illustrating the synergistic effects of DEX and IL-10 during the early period. This supports the expectation that a positive immunological response directed by the sequentially releasing surface can promote early osteogenesis and that indirect cultivation validates the immune-mediated regulatory capacity of the P-DPI sample in the initial stage.

Subsequently, the osteogenesis of osteoblasts was evaluated by directly cultivating the cells on different samples for up to 3 weeks. The experimental

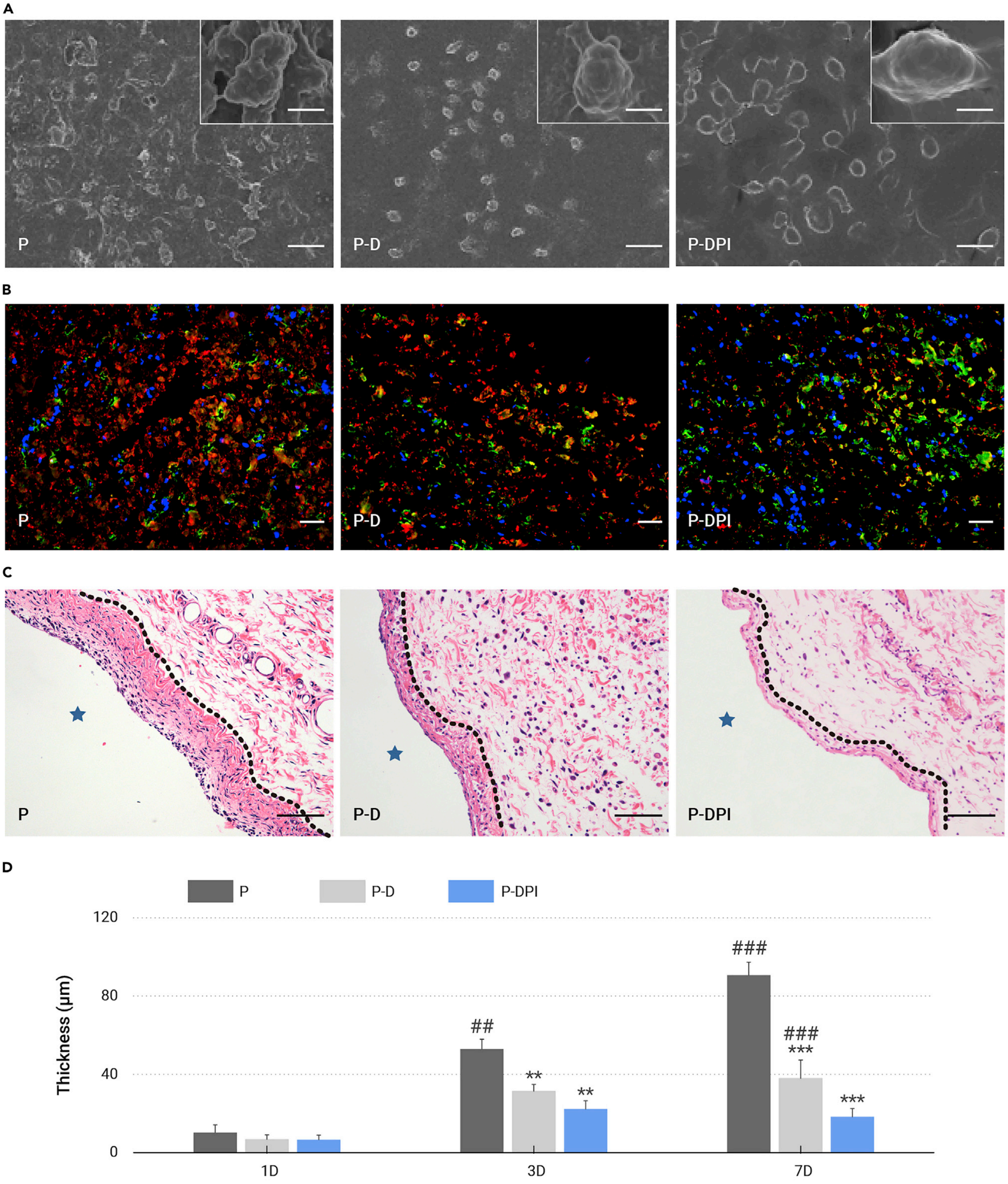

Figure 5. ***In vivo*** **analysis of the inflammatory status** (A) SEM images of macrophages on different samples after implantation for 7 days. (B) Immunofluorescent staining images of macrophages on different samples after implantation for 7 days. Red, green, and blue fluorescence reflect density for iNOS, CD163, and nuclei. (C) H&E staining images of peri-implant tissues after implantation for 7 days. The fibrous layers are marked by dashed lines. (D) Quantitative comparison of fibrous layer thickness after implantation for 1, 3, and 7 days. Scale bars, 100 μm (except those in the insets being equal to 10 μm). **$p < 0.01$ and ***$p < 0.001$ compared with the P group, whereas ##$p < 0.01$ and ###$p < 0.001$ compared with the P-DPI group (n = 6).

design of osteogenic culture is illustrated in Figure 4B, and before which the MC3T3-E1 cells were cultured on different samples without osteogenic induction for 1, 3, and 7 days to evaluate the biocompatibility. As shown in Figure 4D, osteoblasts thrive more on the P-DPI sample than those on the other samples, indicating the excellent surface biocompatibility rendered by DEX and IL-10. Early osteogenesis is represented as ALP activity after osteogenic induction and the P-DPI group is superior to other groups (Figure 4E). Osteogenic differentiation of the directly cultured osteoblasts was also analyzed

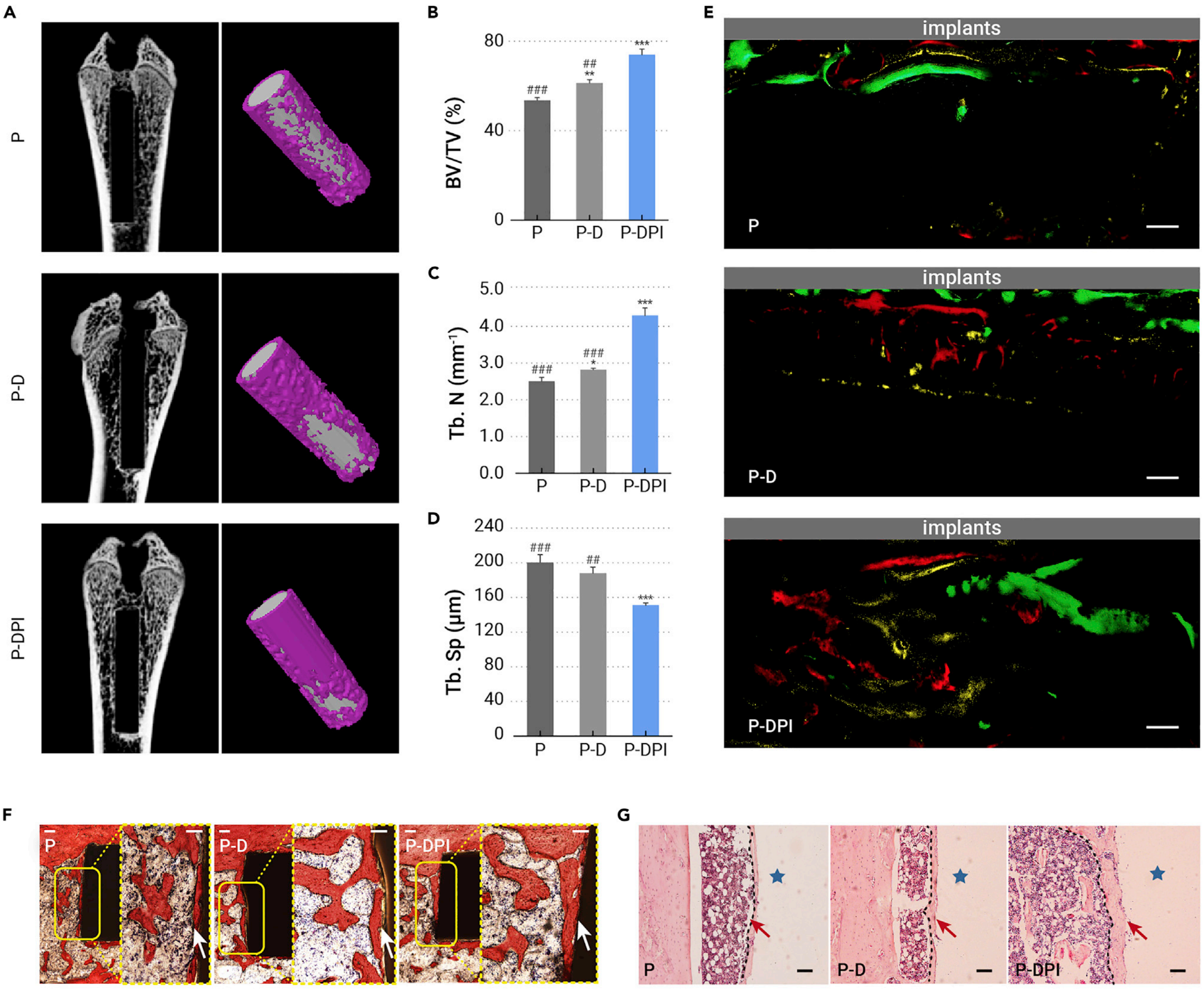

Figure 6. *In vivo* analysis of bone regeneration after implantation for 8 weeks (A) 2D and reconstructed 3D micro-computed tomography (micro-CT) images of the bone with the implants inside. (B–D) Quantitative micro-CT data of (B) bone volume/total volume (BV/TV), (C) trabecular number (Tb.N), and (D) trabecular separation (Tb.Sp). (E) Formation of new bone labeled by sequential fluorescent staining (tissues labeled by alizarin red, tetracycline hydrochloride, and calcein show red, yellow, and green fluorescence, respectively). (F and G) Histological observation of peri-implant tissues after (F) Van Gieson and (G) H&E staining with new bone marked by dashed lines. Scale bars, 100 μm. *$p < 0.05$, **$p < 0.01$, and ***$p < 0.001$ compared with the P group, whereas ##$p < 0.01$ and ###$p < 0.001$ compared with the P-DPI group (n = 6).

in terms of gene expression. Among the modified groups, the expressions of ALP (Figure S8A, supplemental information), OPN (Figure S8B, supplemental information), and OCN (Figure S8C, supplemental information) at all time points were upregulated, with the P-DPI group faring the best. The improved osteogenic differentiation was further verified by the best mineralization status of cells in the P-DPI group (Figure 4F). The results collectively reveal that steady release of DEX followed by IL-10 further enhances the osteogenic effect during the later weeks. All in all, IL-10 and DEX offer synergistic effects to produce the suitable immunological environment for bone formation. Both immune-mediated regulation and direct osteogenic promotion are crucial to the osseointegration of bone implants, and the sequential release of IL-10 and DEX designed in the P-DPI group can well match the bio-progress of bone formation.

Inflammation *in vivo*

In vivo inflammatory responses were analyzed from the morphological, immunofluorescent, and histological perspectives. Under SEM observation, the P-D and P-DPI samples showed a slippery surface after implantation for 1 day, but proteins tended to adhere to the bare PEEK, leading to adherence and proliferation of macrophages (red arrows in Figure S9, supplemental information). After implantation for 7 days, the sporadic macrophages in the P-DPI group showed an elongated shape compared with the spherical ones in the other two groups, indicating M2 polarization consistent with the aforementioned *in vitro* results (Figure 5A).[36] The polarization of macrophages *in vivo* was further determined by immunofluorescent staining of nitric oxide synthase (iNOS as M1 marker) and cluster of differentiation 163 (CD163 as M2 marker). There were many more M2 macrophages detected in the P-DPI group than detected in the P and P-D groups, which validates the positive regulatory effect of P-DPI group (Figures 5B and S10, supplemental information). The inflammatory infiltration peri-implant was observed after hematoxylin and eosin (H&E) staining. The fibrous layer in the P group was the thickest, followed by a thinner layer in the P-D group, and the thinnest in the P-DPI group (Figures 5C and S11, supplemental information). Notably, the thickness of the fibrous layer in the P-DPI group decreased after implantation for 7 days, but those in the other two groups increased gradually (Figure 5D), indicating that DEX and IL-10 work together to relieve inflammation.

Bone formation *in vivo*

Bone formation is the benchmark determining the *in vivo* osteogenic properties, samples were implanted for up to 8 weeks and evaluated systematically. As shown in Figure 6A, the 2D and reconstructed 3D micro-computed tomography scanning images show that peri-implant bone regeneration in the P-DPI group was much better than that in the P and P-D groups. With a bone volume/total volume of 74%, trabecular number of more than 4 mm^{-1}, and trabecular separation of 150 μm (Figures 6B–6D), the P-DPI group possessed the best performance of bone remodeling. The whole osteogenesis process was tracked by sequential fluorescent staining (Figure 6E). Red, yellow, and green fluorescence indicate the new bone formation after implantation for 2, 4, and 6 weeks, respectively, and the total fluorescent area in each group is plotted in Figure S12 (supplemental information). Evidently, the P-DPI group holds the largest fluorescent area, which is 1.8–2.5 times that of the other two groups. Besides the quantity, the quality of new bone was examined from the histological perspective. Van Gieson staining (Figure 6F) and H&E staining (Figure 6G) confirmed that the new bone in the P-DPI group was denser and thicker (white and red arrows, respectively) than that in the P and P-D groups. Notably, the largest ratio of bone-to-implant contact was observed from the P-DPI group (Figure S13, supplemental information). Altogether, the immunological environment created in the first few days and stable DEX release in subsequent weeks led to excellent osteogenesis, as manifested by the quality and quantity of new bone.

Nowadays, the pursuit of implant candidates has delivered PEEK to the site of interest because of the favorable mechanical, as well as chemical stability, and natural radiolucency.[37–39] However, the easily formed fibrous layer on bare PEEK hinders osteointegration, thus raising enthusiasm for various modification strategies.[40–46] Previous studies have mainly concentrated on introducing biocompatible elements into the materials and/or constructing functional coatings, the *in vitro* effects of which are plausible but the lack of systematic consideration of immune-mediated regulation has hampered success *in vivo*. Herein, the immune response is taken into full consideration as hinted by recently proposed theories.[47] Rather than modulating the immune response by adjusting the physical or chemical properties such as the surface roughness/morphology and ion release,[48,49] we deliver the synergistic effect of humanized IL-10 and DEX to minimize immune rejection. As an anti-inflammatory cytokine, the cascade of IL-10 in the first couple of days sends a signal that inflammation is relieved and the M1-M2 transition is underway. A positive response from the immune system is then triggered and macrophages secrete more anti-inflammatory cytokines and inhibit production of pro-inflammatory counterparts, with the corresponding genes being regulated in parallel. At the same time, genes related to autophagy are upregulated and, together with the sustained release of anti-inflammatory DEX, reduce inflammation and promote M2 polarization of macrophages. Collectively, the suitable immunological surroundings are created to promote osteogenesis.

This is the first attempt to program PEEK surface to initiate immune mediation and fulfill bone regeneration sequentially. Our results reveal that this strategy is superior to most other approaches in which the functionalized surfaces only play roles in a certain stage after implantation. In our experiments, PEEK is coated with PTMC containing DEX and subjected to N_2 PIII to facilitate surface grafting of IL-10. The functional molecules are programmed for sequential release that follows the first-order kinetics. Compared with the other biodegradable polymers, PTMC is chosen as the coating material in this study because its degradation via surface erosion is desirable for the sequential release of loaded molecules, and, moreover, the non-acidic products after PTMC degradation contribute to excellent biocompatibility and cause little inflammation.[30,50,51] The cascade of IL-10 and a small amount of DEX creates a suitable immunological environment within 1 week after implantation. By taking advantage of the immunomodulatory effects, osteoblasts thrive on the implant and make a steady transition to the bone-formation stage. In the next few weeks, the stable release of DEX guarantees the quantity and quality of new bone and, consequently, cohesion between the implant and new bone is improved resulting in excellent osseointegration.

It should also be mentioned that previous research activities primarily focus on the functionalization of bone repair materials individually, but the strategies may not be applicable to other regenerative biomaterials. In contrast, this work starts with a comprehensive cognition about different phases of bone regeneration, including immune system activation, polarization of macrophages, differentiation of osteoblasts, bone formation, and bone remodeling, as well as the key factors linking the adjacent phases. The programmed surface is designed to produce a smooth transition through the different phases with minimal side effects and this novel and effective concept can be extended to other prosthetic systems.

Conclusions

A programmed surface was designed and fabricated to achieve immune-mediated osteogenic regulation. In this process, IL-10 and DEX are released sequentially in a specific time window. The cascade of IL-10 and a small amount of DEX in the first few days hinders inflammation and promotes M2 polarization of the surrounding macrophages, creating a suitable immunological environment for bone regeneration. In the ensuing bone-formation stage, steady release of DEX fosters osteogenesis in terms of both quantity and quality. As a result, new bone is formed on the programmed surface via immune-mediated regulation and robust bone-implant osseointegration is obtained. This novel concept and better understanding bode well for success *in vivo* and provide insights into the design of advanced biomedical implants for tissue engineering and immunotherapeutic applications.

REFERENCES

1. Schindler, O.S., Cannon, S.R., Briggs, T.W.R., and Blunn, G.W. (2007). Use of a novel bone graft substitute in peri-articular bone tumours of the knee. The Knee ***14***, 458–464.
2. Cook, E.A., and Cook, J.J. (2009). Bone graft substitutes and allografts for reconstruction of the foot and ankle. Clin. Podiatr. Med. Sur. ***26***, 589–605.
3. Carson, J.S., and Bostrom, M.P.G. (2007). Synthetic bone scaffolds and fracture repair. Injury-int. J. Care Inj. ***38***, S33–S37.
4. Alvarez, M.M., Liu, J.C., Trujillo-de Santiago, G., et al. (2016). Delivery strategies to control inflammatory response: modulating M1–M2 polarization in tissue engineering applications. J. Control Release ***240***, 349–363.
5. Zhuang, H., Wei, F., Jiang, L., et al. (2020). Assessment of spinal tumor treatment using implanted 3D-printed vertebral bodies with robotic stereotactic radiotherapy. The Innovation ***1***, 100040.
6. El-Jawhari, J.J., Jones, E., and Giannoudis, P.V. (2016). The roles of immune cells in bone healing; what we know, do not know and future perspectives. Injury-int. J. Care Inj. ***47***, 2399–2406.
7. Chen, Z., Ni, S., Han, S., et al. (2017). Nanoporous microstructures mediate osteogenesis by modulating the osteo-immune response of macrophages. Nanoscale ***9***, 706–718.
8. Lu, Z., Peng, Z., Liu, C., et al. (2020). Current status and future perspective of immunotherapy in gastrointestinal cancers. The Innovation ***1***, 100041.
9. Xie, Y., Hu, C., Feng, Y., et al. (2020). Osteoimmunomodulatory effects of biomaterial modification strategies on macrophage polarization and bone regeneration. Regen. Biomater. ***7***, 233–245.
10. Chen, Z., Klein, T., Murray, R.Z., et al. (2016). Osteoimmunomodulation for the development of advanced bone biomaterials. Mater. Today ***19***, 304–321.
11. Mehta, M., Schmidt-Bleek, K., Duda, G.N., and Mooney, D.J. (2012). Biomaterial delivery of morphogens to mimic the natural healing cascade in bone. Adv. Drug Deliv. Rev. ***64***, 1257–1276.
12. Liu, R., Chen, S., Huang, P., et al. (2020). Immunomodulation-based strategy for improving soft tissue and metal implant integration and its implications in the development of metal soft tissue materials. Adv. Funct. Mater. ***30***, 1910672.
13. Kohli, N., Ho, S., Brown, S.J., et al. (2018). Bone remodelling in vitro: where are we headed? A review on the current understanding of physiological bone remodelling and inflammation and the strategies for testing biomaterials in vitro. Bone ***110***, 38–46.
14. Bai, L., Du, Z., Du, J., et al. (2018). A multifaceted coating on titanium dictates osteoimmunomodulation and osteo/angio-genesis towards ameliorative osseointegration. Biomaterials ***162***, 154–169.
15. Li, C., Li, Y., Qin, G., et al. (2020). Regulatory role of retinoic acid in male pregnancy of the seahorse. The Innovation ***1***, 100052.
16. Brown, B.N., Ratner, B.D., Goodman, S.B., et al. (2012). Macrophage polarization: an opportunity for improved outcomes in biomaterials and regenerative medicine. Biomaterials ***33***, 3792–3802.

17. Brown, B.N., and Badylak, S.F. (2013). Expanded applications, shifting paradigms and an improved understanding of host–biomaterial interactions. Acta Biomater. ***9***, 4948–4955.
18. Walschus, U., Hoene, A., Neumann, H.-G., et al. (2009). Morphometric immunohistochemical examination of the inflammatory tissue reaction after implantation of calcium phosphate-coated titanium plates in rats. Acta Biomater. ***5***, 776–784.
19. Nathan, K., Lu, L.Y., Lin, T., et al. (2019). Precise immunomodulation of the M1 to M2 macrophage transition enhances mesenchymal stem cell osteogenesis and differs by sex. Bone Jt. Res. ***8***, 481–488.
20. Chen, Z., Bachhuka, A., Han, S., et al. (2017). Tuning chemistry and topography of nanoengineered surfaces to manipulate immune response for bone regeneration applications. ACS Nano ***11***, 4494–4506.
21. Xu, X., Li, Y., Wang, L., et al. (2019). Triple-functional polyetheretherketone surface with enhanced bacteriostasis and anti-inflammatory and osseointegrative properties for implant application. Biomaterials ***212***, 98–114.
22. Wu, C., Chen, Z., Wu, Q., et al. (2015). Clinoenstatite coatings have high bonding strength, bioactive ion release, and osteoimmunomodulatory effects that enhance in vivo osseointegration. Biomaterials ***71***, 35–47.
23. Moore, K.W., de Waal Malefyt, R., Coffman, R.L., and O'Garra, A. (2001). Interleukin-10 and the interleukin-10 receptor. Ann. Rev. Immunol. ***19***, 683–765.
24. Boehler, R.M., Kuo, R., Shin, S., et al. (2014). Lentivirus delivery of IL-10 to promote and sustain macrophage polarization towards an anti-inflammatory phenotype. Biotechnol. Bioeng. ***111***, 1210–1221.
25. Mahon, O.R., Browe, D.C., Gonzalez-Fernandez, T., et al. (2020). Nano-particle mediated M2 macrophage polarization enhances bone formation and MSC osteogenesis in an IL-10 dependent manner. Biomaterials ***239***, 119833.
26. Eijken, M., Koedam, M., van Driel, M., et al. (2006). The essential role of glucocorticoids for proper human osteoblast differentiation and matrix mineralization. Mol. Cell. Endocrinol. ***248***, 87–93.
27. Sher, L.B., Harrison, J.R., Adams, D.J., and Kream, B.E. (2006). Impaired cortical bone acquisition and osteoblast differentiation in mice with osteoblast-targeted disruption of glucocorticoid signaling. Calcified. Tissue Int. ***79***, 118–125.
28. Qian, Y., Li, L., Song, Y., et al. (2018). Surface modification of nanofibrous matrices via layer-by-layer functionalized silk assembly for mitigating the foreign body reaction. Biomaterials ***164***, 22–37.
29. Vyner, M.C., Li, A., and Amsden, B.G. (2014). The effect of poly(trimethylene carbonate) molecular weight on macrophage behavior and enzyme adsorption and conformation. Biomaterials ***35***, 9041–9048.
30. Sommerfeld, S.D., Zhang, Z., Costache, M.C., et al. (2014). Enzymatic surface erosion of high tensile strength polycarbonates based on natural phenols. Biomacromolecules ***15***, 830–836.
31. Schlundt, C., El Khassawna, T., Serra, A., et al. (2018). Macrophages in bone fracture healing: their essential role in endochondral ossification. Bone ***106***, 78–89.
32. Franz, S., Rammelt, S., Scharnweber, D., and Simon, J.C. (2011). Immune responses to implants—a review of the implications for the design of immunomodulatory biomaterials. Biomaterials ***32***, 6692–6709.
33. Marahleh, A., Kitaura, H., Ohori, F., et al. (2019). TNF-alpha directly enhances osteocyte RANKL expression and promotes osteoclast formation. Front. Immunol. ***10***, 2925.
34. Tsukasaki, M., and Takayanagi, H. (2019). Osteoimmunology: evolving concepts in bone-immune interactions in health and disease. Nat. Rev. Immunol. ***19***, 626–642.
35. Xiao, L., and Xiao, Y. (2019). The autophagy in osteoimmonology: self-eating, maintenance, and beyond. Front. Endocrinol. ***10***, 490.
36. Tedesco, S., Bolego, C., Toniolo, A., et al. (2015). Phenotypic activation and pharmacological outcomes of spontaneously differentiated human monocyte-derived macrophages. Immunobiology ***220***, 545–554.
37. Kurtz, S.M., and Devine, J.N. (2007). PEEK biomaterials in trauma, orthopedic, and spinal implants. Biomaterials ***28***, 4845–4869.
38. Gao, A., Liao, Q., Xie, L., et al. (2020). Tuning the surface immunomodulatory functions of polyetheretherketone for enhanced osseointegration. Biomaterials ***230***, 119642.
39. Panayotov, I.V., Orti, V., Cuisinier, F., and Yachouh, J. (2016). Polyetheretherketone (PEEK) for medical applications. J. Mater. Sci.-Mater. Med. ***27***, 118.
40. Dennes, T.J., and Schwartz, J. (2009). A nanoscale adhesion layer to promote cell attachment on PEEK. J. Am. Chem. Soc. ***131***, 3456–3457.
41. Wang, H., Xu, M., Zhang, W., et al. (2010). Mechanical and biological characteristics of diamond-like carbon coated poly aryl-ether-ether-ketone. Biomaterials ***31***, 8181–8187.
42. Shimizu, M., Kobayashi, Y., Mizoguchi, T., et al. (2012). Carbon nanotubes induce bone calcification by bidirectional interaction with osteoblasts. Adv. Mater. ***24***, 2176–2185.
43. Qu, H., Fu, H., Han, Z., and Sun, Y. (2019). Biomaterials for bone tissue engineering scaffolds: a review. RSC Adv. ***9***, 26252–26262.
44. Han, C.-M., Lee, E.-J., Kim, H.-E., et al. (2010). The electron beam deposition of titanium on polyetheretherketone (PEEK) and the resulting enhanced biological properties. Biomaterials ***31***, 3465–3470.
45. Lu, T., Wen, J., Qian, S., et al. (2015). Enhanced osteointegration on tantalum-implanted polyetheretherketone surface with bone-like elastic modulus. Biomaterials ***51***, 173–183.
46. Khoury, J., Selezneva, I., Pestov, S., et al. (2019). Surface bioactivation of PEEK by neutral atom beam technology. Bioact. Mater. ***4***, 132–141.
47. Chen, Z., Visalakshan, R.M., Guo, J., et al. (2019). Plasma deposited poly-oxazoline nanotextured surfaces dictate osteoimmunomodulation towards ameliorative osteogenesis. Acta Biomater. ***96***, 568–581.
48. Refai, A.K., Textor, M., Brunette, D.M., and Waterfield, J.D. (2004). Effect of titanium surface topography on macrophage activation and secretion of proinflammatory cytokines and chemokines. J. Biomed. Mater. Res. **A**. ***70A***, 194–205.
49. Nel, A.E., Maedler, L., Velegol, D., et al. (2009). Understanding biophysicochemical interactions at the nano-bio interface. Nat. Mater. ***8***, 543–557.
50. Zhang, Z., Kuijer, R., Bulstra, S.K., et al. (2006). The in vivo and in vitro degradation behavior of poly(trimethylene carbonate). Biomaterials ***27***, 1741–1748.
51. Athanasiou, K.A., Niederauer, G.G., and Agrawal, C.M. (1996). Sterilization, toxicity, biocompatibility and clinical applications of polylactic acid polyglycolic acid copolymers. Biomaterials ***17***, 93–102.

ACKNOWLEDGMENTS

The authors acknowledge the National Natural Science Foundation of China (nos. 31922040 and 32000962), Shenzhen Science and Technology Research Funding (nos. SGLH20180625144002074 and JCYJ20180507182637685), Guangdong Basic and Applied Basic Research Foundation (no. 2020B1515120078), Youth Innovation Promotion Association of the Chinese Academy of Sciences (nos. 2017416 and 2020353), Shenzhen – Hong Kong Innovative Collaborative Research and Development Program (no. 9240014), City University of Hong Kong Strategic Research Grant (SRG) (no. 7005264), and Hong Kong Research Grants Council (RGC) General Research Funds (GRF) (no. CityU 11205617).

AUTHOR CONTRIBUTIONS

G.W. and H.W. conceived the idea and designed the experiments. H.W. supervised the project. L.X., Y.W., Q.L., S.M., and X.R. carried out the experiments. L.X., G.W., and H.W. analyzed the data with the help of L.T., W.Z., M.G., and H.P. L.X., G.W., P.K.C., and H.W. wrote the manuscript.

DECLARATION OF INTERESTS

The authors declare no competing interests.

SUPPLEMENTAL INFORMATION

Supplemental information can be found online at https://doi.org/10.1016/j.xinn.2021.100148.

LEAD CONTACT WEBSITE

http://htod.siat.ac.cn/Team/detail.shtml?id=4&cid=11.

The Innovation, Volume *2*

Supplemental Information

Programmed surface on poly(aryl-ether-ether-ketone) initiating immune mediation and fulfilling bone regeneration sequentially

Lingxia Xie, Guomin Wang, Yuzheng Wu, Qing Liao, Shi Mo, Xiaoxue Ren, Liping Tong, Wei Zhang, Min Guan, Haobo Pan, Paul K. Chu, and Huaiyu Wang

Supporting Information

Programmed surface on poly(aryl-ether-ether-ketone) initiating immune mediation and fulfilling bone regeneration sequentially

Lingxia Xie,[1,4] Guomin Wang,[2,4] Yuzheng Wu,[1,2] Qing Liao,[1] Shi Mo,[2] Xiaoxue Ren,[1] Liping Tong,[1] Wei Zhang,[3] Min Guan,[1] Haobo Pan,[1] Paul K. Chu,[2] and Huaiyu Wang[1,*]

[1] Center for Human Tissues and Organs Degeneration, Shenzhen Institute of Advanced Technology, Chinese Academy of Sciences, Shenzhen 518055, China

[2] Department of Physics, Department of Materials Science and Engineering, and Department of Biomedical Engineering, City University of Hong Kong, Tat Chee Avenue, Kowloon, Hong Kong, China

[3] Technical Institute of Physics and Chemistry, Chinese Academy of Sciences, Beijing 100190, China

[4] These authors contributed equally

* Correspondence：hy.wang1@siat.ac.cn

Experimental procedure

Preparation and characterization of samples

PEEK was cut into round plates (diameter = 1.5 cm, thickness = 2 mm, *in vitro*) and cylinders (diameter = 1.5 mm, thickness = 7 mm, *in vivo*), respectively. The samples were polished with sandpaper (800, 1200 and 2000 mesh) and treated ultrasonically with acetone, ethanol, and pure water. The coating of PTMC with DEX (5 wt%) was fabricated on PEEK by solvent evaporation. In detail, DEX and PTMC (1:20) was dissolved in dichloromethane and the solution was spread onto the PEEK samples, vacuum dried at room temperature (0.8 Mpa) for 24 hours until a solid film consisting of PTMC and DEX was formed on the surface. In the next step, the samples were subjected to N_2 PIII (pulse frequency = 1000 Hz, duration = 50 μs, and voltage = 2 kV) for 60 minutes. The treated samples were then immersed in IL-10 (40 ng mL^{-1}) for 24 hours for grafting and then treated with phosphate buffered saline (PBS) to remove loose IL-10. Control and experimental groups are presented as PEEK (P), PEEK + PTMC/DEX coating (P-D), PEEK + PTMC/DEX coating + N_2 PIII (P-DP), and PEEK + PTMC/DEX coating + N_2 PIII + IL-10 grafting (P-DPI).

The surface morphology was examined by SEM (ZEISS SUPRA 55, Germany). Surface hydrophilicity was determined by stastic contact angle measurement (Attension Theta Flex, Biolin Scientific, Sweden) using 4 µL of sessile distilled water under ambient conditions. The surface chemical states were determined by XPS (ESCALAB350Xi, Thermo Fiser, USA) with Al K_α radiation referenced to the Ar 2p peak at 242.4 eV. The adhesion of coating on PEEK substrate was studied using a scratch tester (WS-2005, Zhongke Kaihua Technology, China), with the load ranging from 0 N to 20 N at a speed of 3 mm min^{-1} and 50 N min^{-1}.

Releasing kinetics of IL-10 and DEX

To evaluate the release kinetics of IL-10 and DEX, the P-DPI samples with the hybrid coating (n = 4 per group) were incubated in a solution containing lipase and maintained on a

constant temperature oscillator (37 ˚C) for up to 28 days. The samples were harvested after 1, 3, 5, 7, 14, 21 and 28 days, rinsed with PBS, dried in the air, and dissolved in dichloromethane for elution. The concentrations of IL-10 and DEX in dichloromethane were tested by enzyme-linked immuno sorbent assay (ELISA) using a Mouse IL-10 DuoSet ELISA kit (R&D Systems, USA) and UV-visible spectrophotometry (UV-US, TU-1810, Pulse, China), respectively. The release rates were determined by reverse calculation.

Cell cultivation

Murine-derived macrophages (RAW 264.7) and osteoblasts (MC3T3-E1 cells) from the American Type Culture Collection (ATCC) were utilized in our study and culture at 37 °C. The RAW264.7 cells were maintained in the high-glucose dulbecco's minimum essential medium (DMEM, Hyclone, USA) and the MC3T3-E1 cells were cultured in the alpha minimum essential medium (α-MEM, Hyclone, USA). Fetal bovine serum (10%, Gibco, USA) and penicillin/streptomycin (1% v/v, Invitrogen, USA) were added. All the samples were irradiated by UV for half an hour before cell seeding.

Assessment on RAW 264.7 cells

Proliferation. Samples after sterilization were seeded with 2×10^4 RAW 264.7 cells per sample. After incubating for 1, 3 and 5 days, CCK-8 (Beyotime, China) was used to evaluate cell proliferation. Samples with the cells were then harvested, washed with PBS for three times, and dispersed in 400 μL of culture medium containing 10% CCK-8. After incubation for another 1 hour at 37 °C, the medium was mixed thoroughly and the supernatant (200 μL) was collected to test OD450 using a microplate reader (BL340, Biotech, USA).

Morphology. After incubating for 1, 3 and 5 days, the cells were observed by SEM. For preparation, samples were washed with PBS for three times and then immersed in 2.5% glutaraldehyde for fixation. The fixed samples were sequentially treated with ethanol (30%, 40%, 50%, 70%, 90% and 100% v/v) for dehydration, dried in air, and sputtered with platinum.

Inflammatory factors. After incubating for 1 and 3 days, the expressions of TNF-α and TGF-β1 were examined by ELISA. Supernatant was centrifuged at 2000 × g for 5 minutes and the mouse TGF-β1 Valukine ELISA Kit (R&D, USA) and mouse TNF-α Valukine ELISA Kit (R&D, USA) were used following the manufacturer's instruction. In each assay, the concentration of inflammatory factors was determined refering to the standard curve, the total cell protein in each group is measured by using a bicinchoninic acid protein assay kit (Beyotime, China) for normalization.

Macrophages phenotypes. The phenotypes of macrophages were determined by detecting CCR7 (M1 marker) and CD206 (M2 marker) on cell membrane, respectively. In particular, the cells were trypsinized after being cultured on various samples for 3 days, centrifuged at 1500 r/min for 5 minutes, and rinsed with PBS. The harvested cells were treated with CCR7 (R&D Systems, USA) and CD206 (R&D Systems, USA) antibodies for 30 minutes at 4 ˚C, tested by flow cytometry (Beckman CytoFLEXS, USA).

In a next step, the phenotypes of macrophages were detected in terms of gene expression. After cultivating on various samples for 3 days, culture medium was collected and centrifuged at 1500 r/min (5 min) before the supernatants were collected as the conditioned media for the following experiments. The RNA in the cultured cells was extracted by trizol reagent (Qiagen, Hilden, Germany) and reversely transcribed into cDNA (RevertAid First Strand cDNA Synthesis Kit, Thermo Fisher). The expressions of CCR7, TNF-α, IL1-β and INOS for M1 phenotype and CD206, TGF-β1, BMP-2 and VEGF for M2 phenotype were analyzed by RT-PCR (Bio-Rad CFX 96, Transgen Biotech, China). The forward and reverse primers were shown in Table S1. Furthermore, the gene expressions related to autophagy including ATG7, LC3A,LC3B and P62 were quantitatively determined by RT-PCR as described above.

Osteogenesis of MC3T3-El cells cultivated in conditioned medium

To investigate whether the macrophages could influnce the osteogenesis process, RAW 264.7 cells were firstly cultured on different samples for 1, 2 and 3 days and the medium were collected. Afterwards, the collected medium was mixed with an equal volume of the normal culture medium to compose the conditioned medium for culturing MC3T3-E1 cells. For the RAW 264.7 (-) groups, the different samples without cell seeding were immersed in the medium for up to 3 days, and the medium after immersion was collected to compose the conditioned medium. The MC3T3-E1 cells were cultivated in the normal culture medium for 12 hours before replacing the medium with the conditioned medium. After cultivation for another 3 days, the cells after rinsing were treated with the cell lysis buffer (Beyotime, China). The ALP activity was evaluated by the ALP assay kit (Beyotime, China) and normalized to total intracellular protein amount tested by the bicinchoninic acid protein assay kit (Beyotime, China). The RNA in different groups was subjected to RT-PCR to quantitatively evaluate the osteogenic gene expressions including ALP, OPN and OCN.

Osteogenesis of MC3T3-E1 directly cultivated on samples

After cultavation for 1, 3 and 7 days, proliferation of cells was tested by CCK-8 assay as described above. Furthermore, MC3T3-E1 proliferated on different samples for 3 days was subjected to osteogenic induction by refreshing the culture medium and supplementing with ascorbic acid (50 μg ml^{-1}) and β-glyceryl phosphate (10 mM). 3 and 7 days later, cells on samples were quantitatively analyzed for the ALP activity following the protocols stated above. The ALP was also stained by the BCIP/NBT kit (Beyotime, China) for observation. After induction for 7, 14 and 21 days, gene expression of ALP, OPN and OCN was again evaluated by RT-PCR as described above.

The mineralization state of extracellular matrix was stained by alizarin red (Beyotime, China). After osteogenic induction for 14 and 21 days, samples were taken out, rinsed with PBS for three times, and fixed with 75% ethanol for 1 hour. Afterwards, all the specimens reacted with alizarin red (40 mM, pH = 4.2) for 30 minutes. Distilled water flushing was then

used to remove the unbound stain and bound stain was dissolved by adding 500 μL cetylpyridinium chloride (10%, pH = 7.0). 200 μL of the solution in each group was then used to test OD570 by microplate reader (BL340, Biotech, USA).

Immune-mediated regulation and osteogenesis *in vivo*

3 months old SD rats (male, 200-300g) were maintained under specific pathogen free (SPF) conditions. The animal experiments in this work were approved by the Ethics Committee for Animal Research of Shenzhen Institute of Advanced Technology, Chinese Academy of Sciences.

Immunological assessments after subcutaneous implantation. The experimental animals were anesthetized with 2% sodium pentobarbital (2.3 mL kg^{-1}) before the samples in different groups were implanted subcutaneously. The implantation sites were symmetrically located on both sides of the dorsal midline. The rats were sacrificed after 1, 3 and 7 days and the samples as well as the surrounding tissues were collected, immersed in paraformaldehyde (4%) for fixation and treated with gradient ethanol for dehydration before SEM observation. The M1 to M2 polarization was determined by staining with the corresponding markers (INOS for M1 and CD163 for M2, Servicebio, China). The nuclei of cells were also stained by 4',6-diamidino-2-phenylindole (DAPI, Servicebio, China) before the observation under fluorescent microscopy (Nikon Eclipse CI, Japan). Afterwards, the specimens were embedded with paraffin, sectioned into slices (5 mm in thickness), and subjected to H&E staining to visualize the tissues surrounding the implants.

Osteogenic assessments. The rats were anesthetized with 2% sodium pentobarbital (2.3 mL kg^{-1}) before defects in cylindrical shape were established at the intercondylar notch of the distal femur. The samples in different groups were implanted into the defects and the wounds were carefully sutured. To monitor the peri-implant new bone formation and mineralization, 3 different fluorochromes including alizarin red (30 mg kg^{-1}, Sigma-Aldrich, USA), tetracycline hydrochloride (25 mg kg^{-1}, Sigma-Aldrich, USA), and calcein (20 mg kg^{-1}, Sigma-Aldrich,

USA) were intraperitoneally administered into the rats after implantation for 2, 4 and 6 weeks, respectively. All the rats were sacrificed 8 weeks later. The femurs with implants were harvested and fixed with 4% paraformaldehyde. Micro-CT (SkyScan 1176, Bruker, Germany) was employed to image the newly formed bone with the 3D images reconstructed (Ctvol, Skyscan). By 3D bone morphometric analysis, BV/TV, Tb.N and Tb.Sp in different groups were determined.

After micro-CT scanning, the fixed samples were dehydrated, embedded, sectioned and ground to a thickness of about 50 μm before the fluorescent observation under confocal laser microscopy (TCS SP8, Leica, Germany). The excitation/emission wavelengths were set at 543/620, 405/575, and 488/520 nm for red, yellow and green fluorescence, respectively. The sections were then subjected to VG staining and H&E staining for the observation of bone-implant interfaces.

Statistical analysis

The experiments in this work were performed in triplicate at leastwith the data being shown as mean ± standard deviation. Statistical evaluation was performed using SPSS software, and differences among groups were determined by analysis of variance (ANOVA) with Bonferroni test. Significant difference was defined at $p < 0.05$ and highly significant difference was defined at $p < 0.01$ or $p < 0.001$.

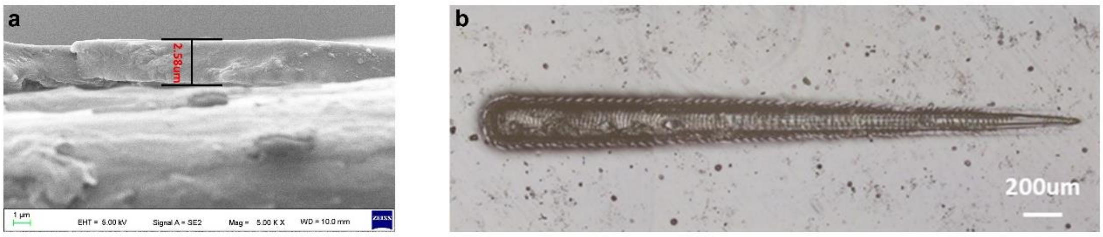

Figure S1: (a) The thickness and (b) scratch test of fabricated coating.

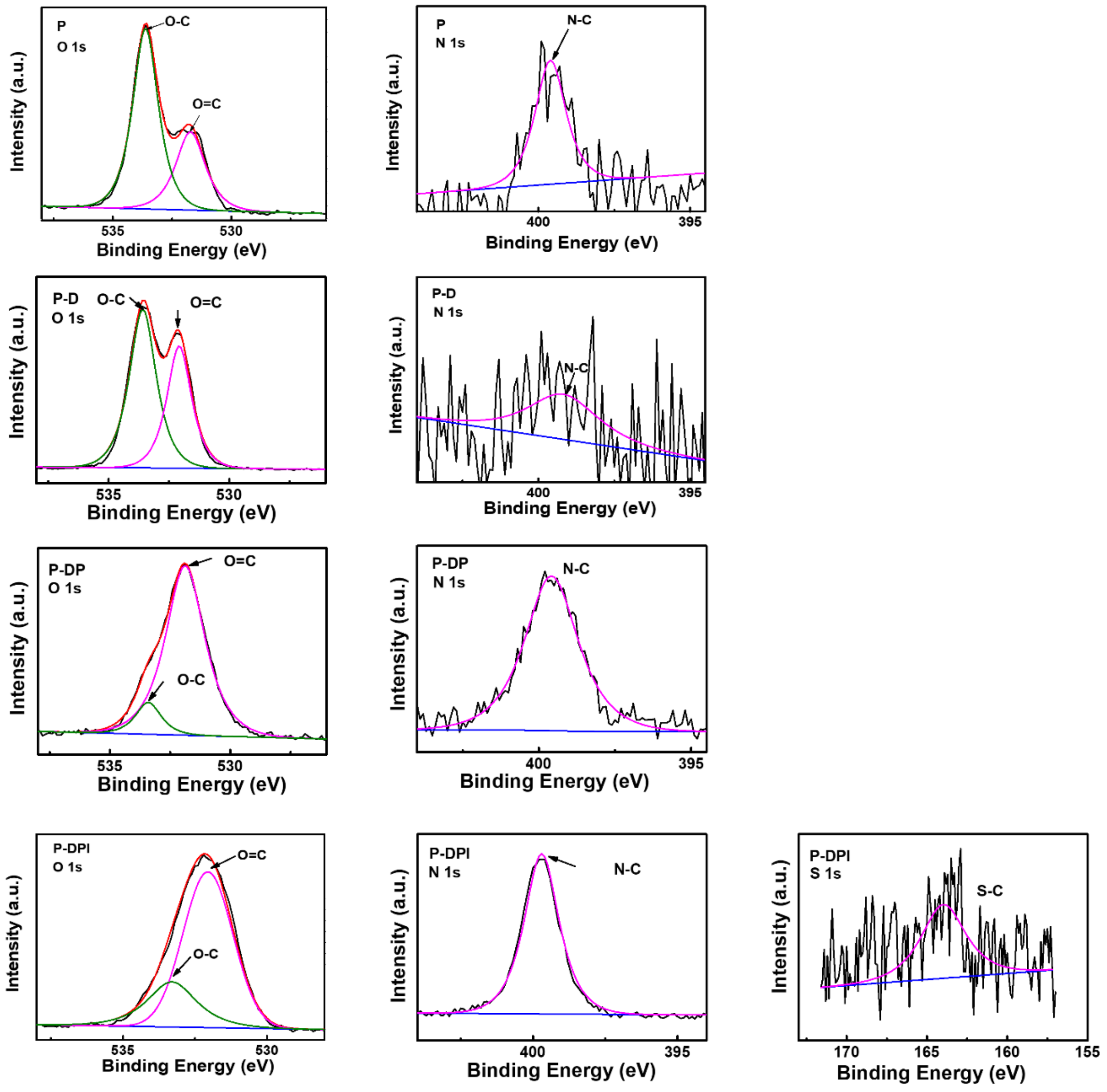

Figure S2: High-resolution XPS spectra for O 1s, N 1s and S 1s acquired from different samples.

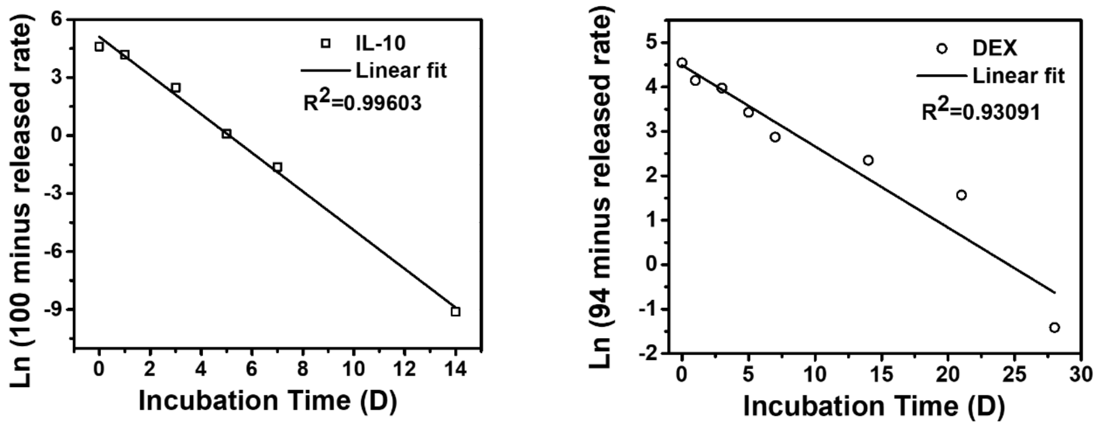

Figure S3: Fitted lines illustrating the relationship between the released rate of IL-10 and DEX on semi-log scale and incubation time.

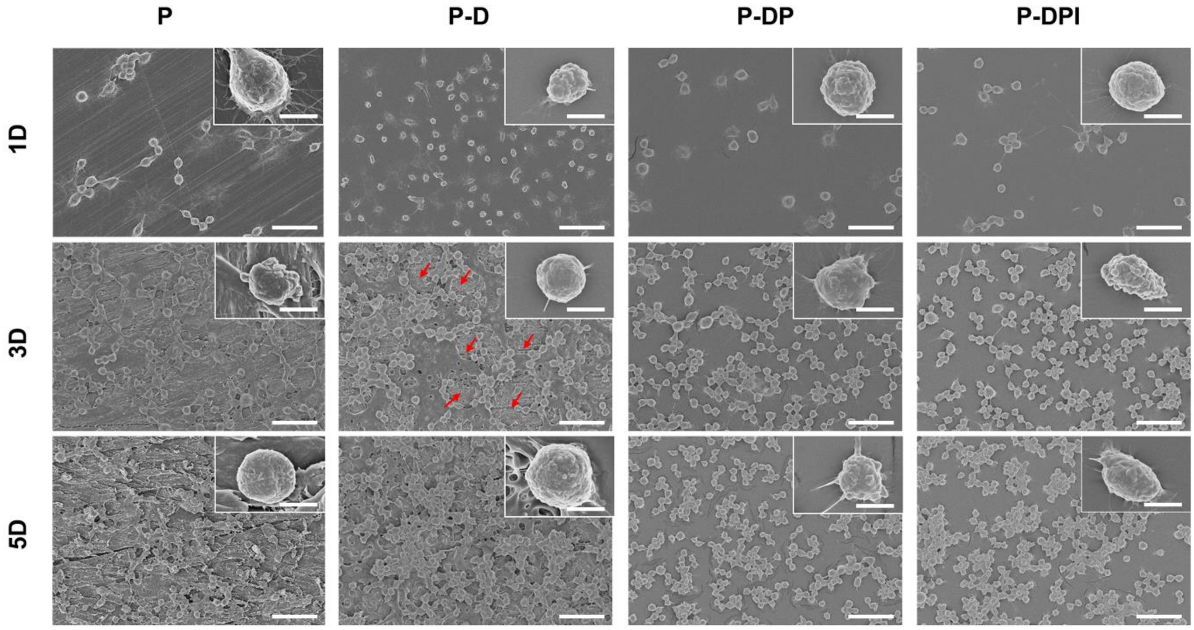

Figure S4: SEM images (scale bar = 35 μm) of macrophages cultivated on samples for 1, 3 and 5 days with insets showing the magnified images (scale bar = 5 μm).

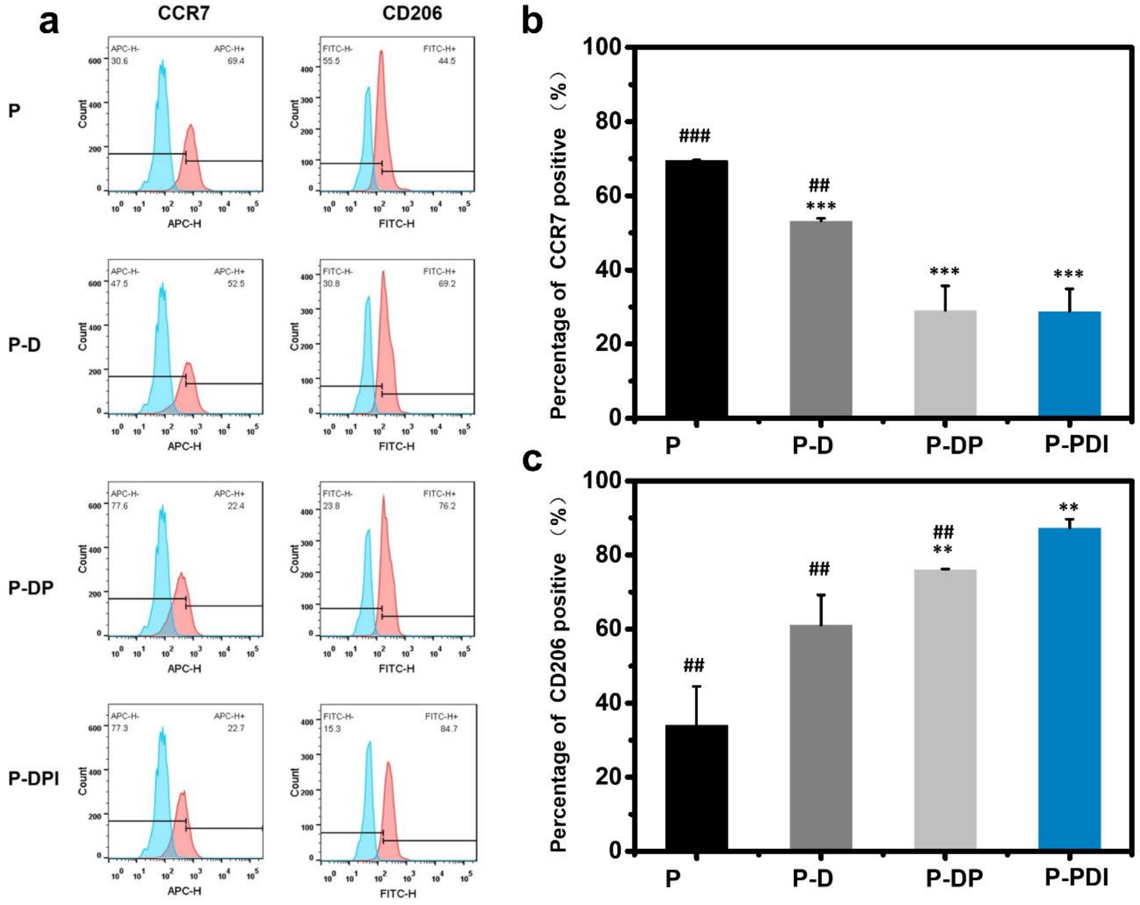

Figure S5: (a) Flow cytometric analysis of RAW264.7 cells cultivated on samples for 3 days; (b) Percentage of CCR7-positive macrophages (M1 phenotype); (c) Percentage of CD206-positive macrophages (M2 phenotype). ** denotes p < 0.01 and *** denotes p < 0.001 compared with the P group, whereas ## denotes p <0.01 and ### denotes p < 0.001 compared with the P-DPI group (n=4).

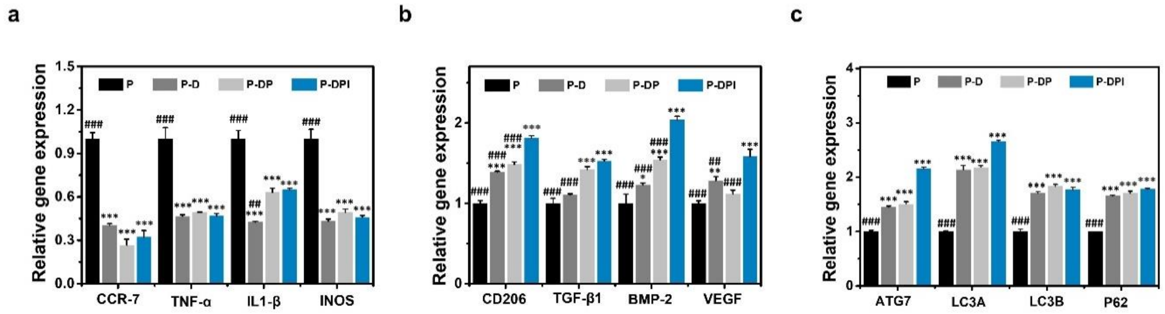

Figure S6: Expression of (a) M1 and (b) M2 genes, and (c) Autophagy-related genes after cultivating macrophages on different samples for 1 day. * denotes p < 0.01** denotes p < 0.01

and *** denotes $p < 0.001$ compared with the P group, whereas ## denotes $p <0.01$ and ### denotes $p < 0.001$ compared with the P-DPI group (n=4).

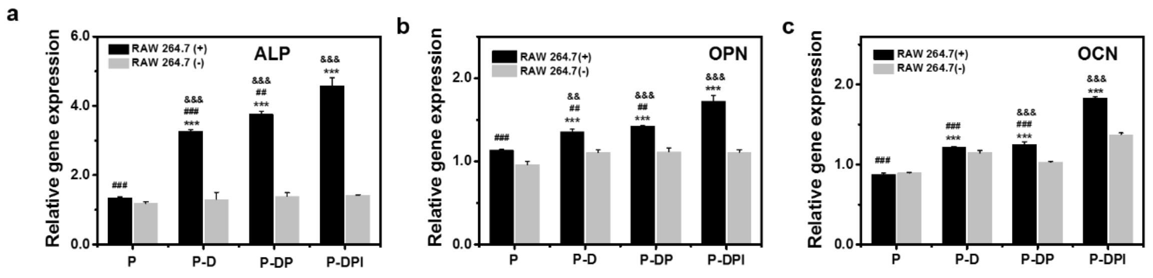

Figure S7: Osteogenic gene expressions of osteoblasts in different groups after conditioned culture: (a) ALP, (b) OPN and (c) OCN. *** denotes $p < 0.001$ compared with the P group, ## denotes $p < 0.01$ and ### denotes $p < 0.001$ compared with the P-DPI group, whereas && denotes $p < 0.01$ and &&& denotes $p < 0.001$ by comparing RAW 264.7 (+) with RAW 264.7 (-) in each group (n=4).

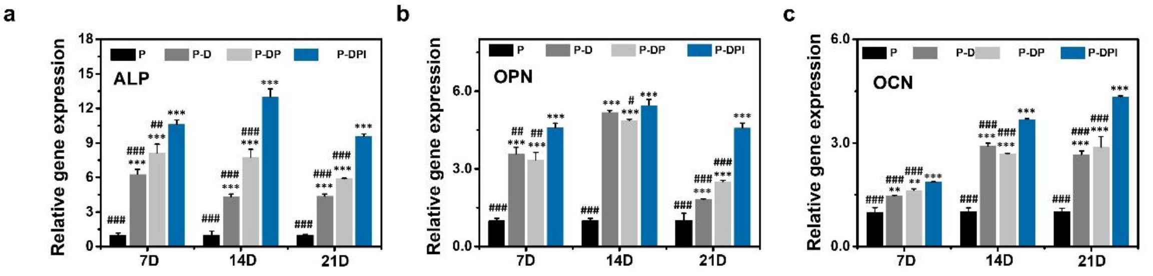

Figure S8: Osteogenic gene expressions of osteoblasts directly cultured on different samples after osteogenic induction for 7, 14 and 21 days: (a) ALP, (b) OPN and (c) OCN. **denotes $p < 0.01$ and *** denotes $p < 0.001$ compared with the P group, whereas # denotes $p <0.05$, ## denotes $p <0.01$ and ### denotes $p < 0.001$ compared with the P-DPI group (n=4).

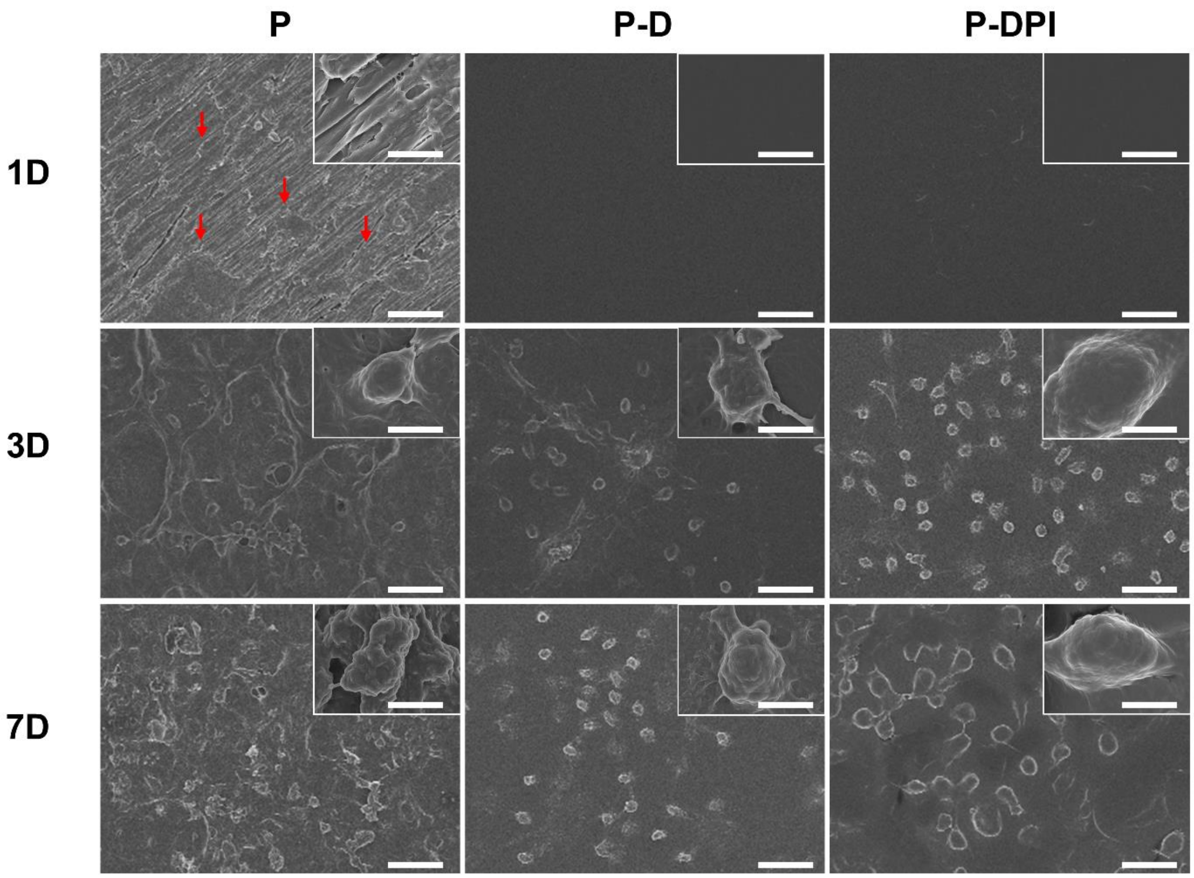

Figure S9: SEM images of macrophages on samples after implantation for 1, 3 and 7 days with insets showing the magnified images (scale bars equal to 100 μm and 10 μm, respectively).

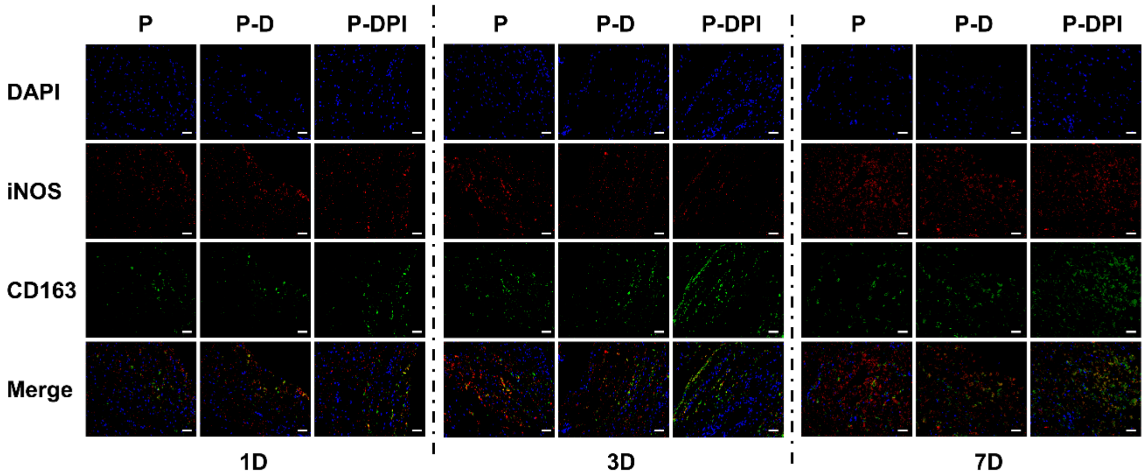

Figure S10: Immunofluorescent staining images of macrophages after implantation for 1, 3 and 7 days: red (INOS for M1), green (CD163 for M2) and blue (nuclei) (scale bar = 100 μm).

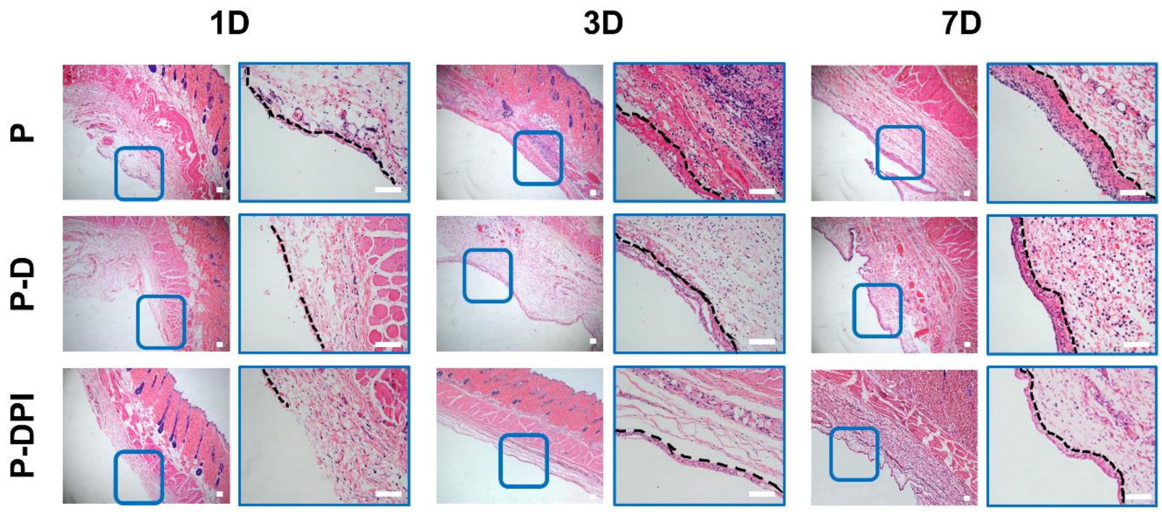

Figure S11: H&E staining images of peri-implant tissues after implantation for 1, 3 and 7 days. The fibrous layers are highlighted by dashed lines (scale bar = 100 μm).

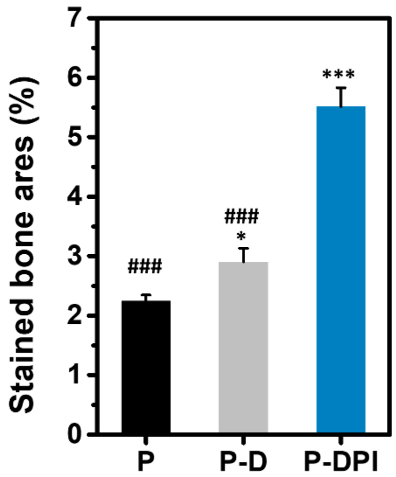

Figure S12: Percentages of stained bone areas in different groups after implantation for 8 weeks. * denotes p <0.05 and *** denotes p <0.001 compared with the P group, whereas ### denotes p < 0.001 compared with the P-DPI group (n=6).

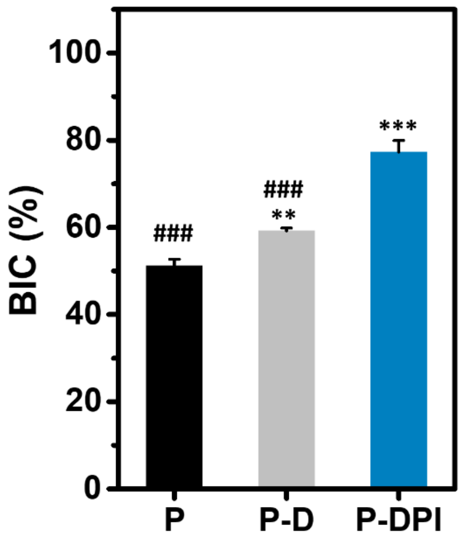

Figure S13: Ratios of BIC in different groups after implantation for 8 weeks. ** denotes p <0.01 and *** denotes p <0.001 compared with the P group, whereas ### denotes p < 0.001 compared with the P-DPI group (n=6).

Table S1. Primers used in RT-PCR.

Primers	Sequences (5'-3')
β-actin	Forward:CGTAAAGACCTCTATGCCAACA
	Reverse: AGCCACCAATCCACACAGAG
CCR7	Forward: GGTGGCTCTCCTTGTCATTTTC
	Reverse: AGGTTGAGCAGGTAGGTATCCG
TNF-α	Forward: TAGCCCACGTCGTAGCAAAC
	Reverse: TGTCTTTGAGATCCATGCCGT
IL-1β	Forward:TGCCACCTTTTGACAGTGATG
	Reverse: GAAGGTCCACGGGAAAGACA
INOS	Forward: GGTGAAGGGACTGAGCTGTTA
	Reverse: TGAAGAGAAACTTCCAGGGGC
CD206	Forward:GCACTGGGTTGCATTGGTTT

	Reverse: CCTGAGTGGCTTACGTGGTT
TGF-β1	Forward:CAGTACAGCAAGGTCCTTGC
	Reverse: ACGTAGTAGACGATGGGCAG
BMP-2	Forward:GCACTGGGTTGCATTGGTTT
	Forward:GGGAAGCAGCAACACTAGAAGA
VEGF	Forward:GTCCCATGAAGTGATCAAGTTC
	Reverse:TCTGCATGGTGATGTTGCTCTCTG
ATG7	Forward:AGCCTGTTCACCCAAAGTTC
	Reverse: CATGTCCCAGATCTCAGCAG
LC3A	Forward:ACAGCATGGTGAGCGTCTC
	Reverse: AGGTTTCTTGGGAGGCGTAG
LC3B	Forward:GATAATCAGACGGCGCTTGC
	Reverse: TCTCACTCTCGTACACTTCGG
P62	Forward:AGCTGCTCTTCGGAAGTCAG
	Reverse: CTCCATCTGTTCCTCTGGCTG
ALP	Forward: TCAGAAGCTAACACCAACG
	Reverse: TTGTACGTCTTGGAGAGGGC
OPN	Forward:TCACCTGTGCCATACCAGTTAA
	Reverse: TGAGATGGGTCAGGGTTTAGC
OCN	Forward: GCAAAGGTGCAGCCTTTGTG
	Reverse: GGCTCCCAGCCATTGATACAG
